# Integrated analysis reveals the participation of IL4I1, ITGB7, and FUT7 in reshaping the TNBC immune microenvironment by targeting glycolysis

**DOI:** 10.1080/07853890.2021.1937694

**Published:** 2021-06-16

**Authors:** Tao Xu, Jiahao Liu, Yu Xia, Zhi Wang, Xingrui Li, Qinglei Gao

**Affiliations:** a Key Laboratory of the Ministry of Education, Cancer Biology Research Center, Tongji Hospital, Tongji Medical College, Huazhong University of Science and Technology, Wuhan, China; b Department of Thyroid and Breast Surgery, Tongji Hospital, Tongji Medical College, Huazhong University of Science and Technology, Wuhan, China; c Department of Obstetrics and Gynecology, Tongji Hospital, Tongji Medical College, Huazhong University of Science and Technology, Wuhan, China

**Keywords:** Triple-negative breast cancer, immunotherapy, glucose metabolism, FDG-PET, PD-(L)1

## Abstract

**Background:**

The overall response rate of immunotherapy in triple-negative breast cancer (TNBC) remains unsatisfactory. Accumulating evidence indicated that glucose metabolic reprogramming could modulate immunotherapy efficacy. However, transcriptomic evidence remains insufficient.

**Methods:**

Genes' relationship with glucose metabolism and TNBC-specific immune was demonstrated by weighted gene co-expression network analysis (WGCNA). The glucose metabolic capability was estimated by standardised uptake value (SUV), an indicator of glucose uptake in 18 F-fluorodeoxyglucose positron emission tomography (FDG-PET), and a reflection of cancer metabolic behaviour. PD-(L)1 expression was used to reflect the efficacy of immunotherapy. Additionally, immune infiltration, survival, and gene coexpression profiles were provided.

**Results:**

Comprehensive analysis revealing that IL4I1, ITGB7, and FUT7 hold the potential to reinforce immunotherapy by reshaping glucose metabolism in TNBC. These results were verified by functional enrichment analysis, which demonstrated their relationships with immune-related signalling pathways and extracellular microenvironment reprogramming. Their expressions have potent positive correlations with Treg and Macrophage cell infiltration and exhausted T cell markers. Meanwhile, their overexpression also lead to poor prognosis.

**Conclusion:**

IL4I1, ITGB7, and FUT7 may be the hub genes that link glucose metabolism, and cancer-specific immunity. They may be potential targets for enhancing ICB treatment by reprogramming the tumour microenvironment and remodelling tumour metabolism.

## Introduction

Triple-negative breast cancer (TNBC) refers to breast cancers not expressing the oestrogen receptor (ER), the progesterone receptor (PR), or human epidermal growth factor receptor 2 (HER2) [1]. Representing for about 15% of all breast cancer cases, TNBC is associated with a dreadful prognosis and a substantial unmet medical need [2]. In the past few years, immune checkpoint blockers (ICB) led to a significant paradigm shift in oncology treatment, which may also offer promise of clinical benefits for TNBC [3]. However, durable responses to immunotherapy were only observed in a small subset of patients [4]. Thus, unlocking the therapeutic potential of ICB and expanding its role in TNBC treatment is of utmost clinical importance.

The treatment efficacy of ICB is largely dependent on the tumour microenvironment and immune regulators [3]. Simultaneously, accumulating evidence showed that glucose metabolism contributes to both factors mentioned above substantially [5]. In the tumour immune microenvironment, accelerated glucose catabolism plays a critical role in suppressing anti-tumour T cell functions by causing glucose deprivation [6]. Moreover, increased glucose consumption and lactate production also lead to defective tumour immunosurveillance [7]. With regard to immune modulate effects, previous studies shed light on the elaborate connection between glucose metabolism and PD-(L)1 (programmed death receptor-1, programmed death receptor ligand-1) expression [8]. On the one hand, elevated glycolytic intensity leads to PD-L1 overexpression *via* PI3K–Akt–mTOR pathway activation, resulting in immune evasion and cancer progression [6]. On the other hand, glucose metabolic deterioration potentially partakes in altered glycosylation of PD-(L)1, which is critical for maintaining its protein stability and cell surface localisation [9–11]. Meanwhile, the capacity of glucose uptake is a crucial hallmark for cancer itself [12]. In 2020, Yue Gong and his college profiled the metabolic dysregulation in TNBC and divided TNBC into metabolic-pathway-based subtypes (MPS) [13]. According to their research, the MPS2 subtype is characterised by upregulated carbohydrate metabolism and exhibited the worst prognosis. Additionally, the standardised uptake value (SUV), as an indicator of glucose uptake in 18 F-fluorodeoxyglucose positron emission tomography (FDG-PET), is closely related to the malignant phenotype of breast cancers [14,15].

Despite the advances mentioned above, our knowledge of interactions between anti-tumour immunity and glucose metabolism remains relatively limited. In this study, insights connecting these two processes are revealed by conducting comprehensive bioinformatic analysis in TNBC. Genes identified in this study could be the nodal hub of the immunometabolism network and future target to reinforce ICB treatment efficacy.

## Methods

### Data collection

The mRNA expression data and SUV assessed by FDG-PET in 84 TNBC patients were downloaded from Gene Expression Omnibus (GEO) database, GSE135565 dataset (http://www.ncbi.nlm.nih.gov/geo/) [14]. RNA-Seq information for 146 TNBC and 111 normal tissues were downloaded from NCI's Genomic Data Commons the Cancer Genome Atlas cohort (TCGA) (https://gdc.cancer.gov/).

### Gene co-expression network construction

The intensity of glucose uptake was measured by SUV, and the expression of PD-(L)1 was considered as a signature of the cancer-associated immunity. WGCNA was performed to identify gene modules correlated with a high level of SUV and PD-(L)1 expression [16]. In this study, WGCNA was performed using R software (version 3.6.2). In the WGCNA algorithm, gene significance (GS, defined as the correlation between the gene and the standardised uptake value) and module membership (MM, defined as the correlation between the gene and its module) were used to quantify the configurations of modules and features.

### Functional enrichment and protein-protein interaction network analysis

The GO (Gene Ontology) annotation and KEGG (Kyoto Encyclopaedia of Genes and Genomes) pathway enrichment analysis for targeted genes were accomplished by the R packages “clusterProfiler” and “enrichplot”. GSEA software was used to examine the different pathways related to target genes. STRING database was used to describe the PPI (protein-protein interaction) network [17].

### Prognosis analysis

PrognoScan allows conducting systematic meta-analysis for prognosis on several GEO datasets. It is capable of evaluating the relationship between gene expression levels and various endpoints, including disease-specific survival (DSS), relapse-free survival (RFS), and overall survival (OS) [18].

### Immune cell infiltration analysis

The infiltration of immune cells, including CD4+ T cells, CD8+ T cells, B cells, neutrophils, macrophages, and dendritic cells, was estimated using the TIMER (Tumour IMmune Estimation Resource) algorithm, basing on RNA-Seq expression profile data of identical immune markers [19]. The correlation between hub genes and immune cells abundance was calculated using Spearman's correlation analysis with correlation coefficient >0.3 indicates a positive/negative correlation. The Gene Expression Profiling Interactive Analysis (GEPIA) database (http://gepia.cancer-pku.cn) was used to analyse the relationship between the expression of hub genes and tumour-infiltrating immune cells marker genes [T cells, TAMs, M1 macrophages, M2 macrophages, T-helper (Th) cells, T-helper 17 (Th17) cells, follicular helper T (Tfh) cells, exhausted T cells, and Tregs], which were identified according to previous studies [20].

### Statistical analysis

Differential analysis of RNA expression profile was performed with R (version 3.6.2). All data were standardised and analysed using the “limma” package. Differential genes were screened according to (corrected *p* < .05, |log FC| >1). Data obtained from TIMER and PrognoScan were presented as HR and *p* values or Cox P values upon the log-rank test. *p* < .05 is considered to be of statistical significance.

## Results

### Identification and annotation of genes related to TNBC-specific glucose metabolism

**Figure 1.**
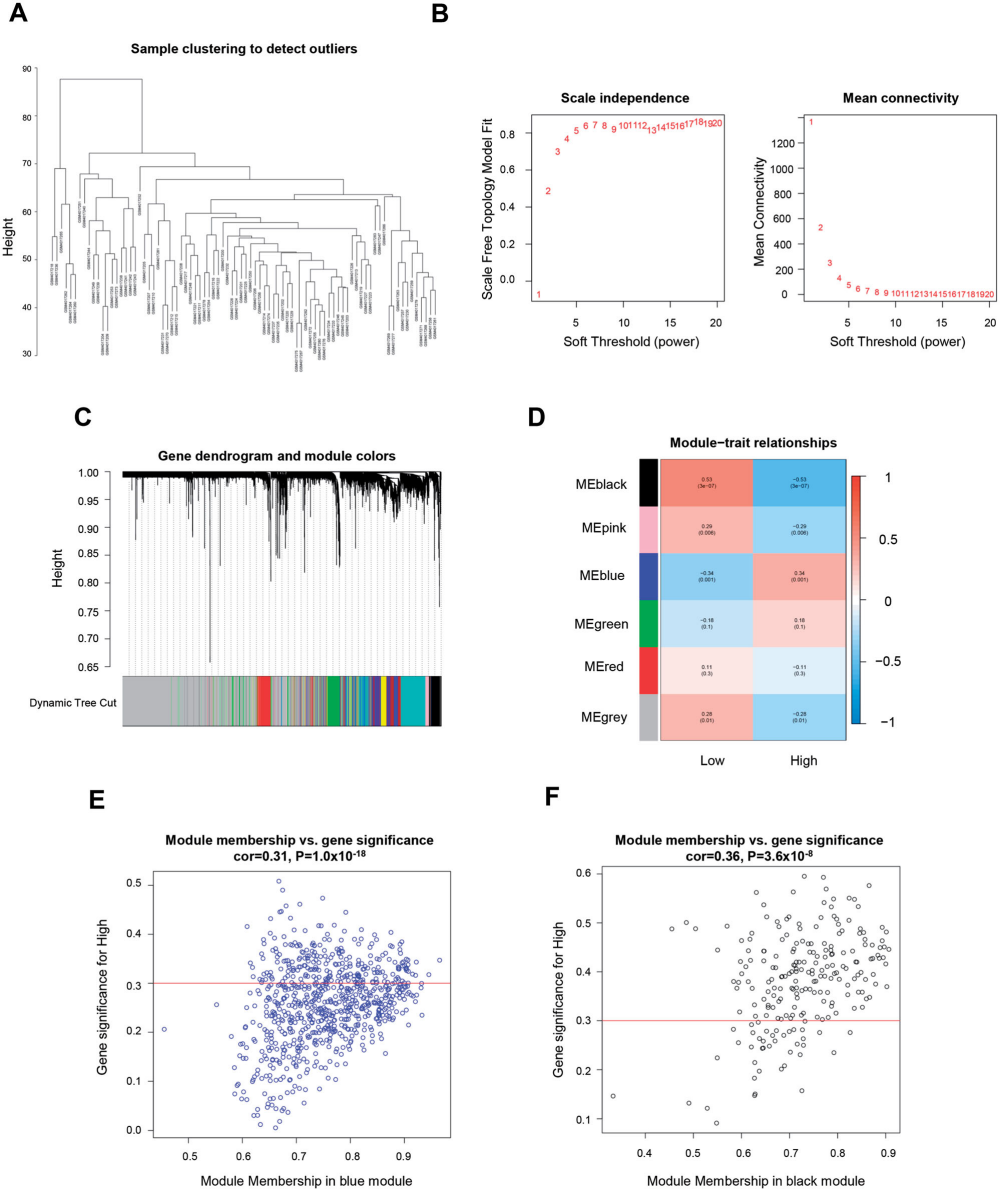
Identification of genes related to TNBC-specific glucose metabolism. (A) Sample clustering to detect outliers. (B) Determination of soft threshold by evaluating the scale-free topology fit index (left) and mean connectivity (right). (C) Dendrogram plot with colour annotation. (D) Heatmap for the correlations of gene modules to SUV. Correlations between module membership and gene significance values were presented in scatterplots for the blue (E) and black (F) gene modules. TNBC: Triple-negative breast cancer; SUV: standardised uptake value.

Gene expression profiles and SUV assessments of 84 TNBC patients were obtained from the GEO database (GSE135565). Patients were divided into either glucose uptake high (GUH) group (SUV ≥ 4.6) or glucose uptake low (GUL) group (SUV<4.6) [14]. WGCNA (Weighted gene co-expression network analysis) was conducted to identify differential gene modules between two groups. Genes with similar expression peculiarities established the same module (Figure 1(A)). The soft threshold β value equalled 18 when the co-expression network satisfied scale-free topology (Figure 1(B)). Six major gene modules were constructed using the average linkage hierarchical clustering (Figure 1(C)). Their correlations with SUVs were evaluated (Figure 1(D)). The upregulation of genes within the blue module has the strongest positive correlation with the GUH group. On the contrary, genes in module black have a significant relationship with the GUL group.

In these gene modules, the hub genes referred to those with MM >0.3 and GS >0.3. As a result, 246 in the blue modules (Figure 1(E); Table S1) and 180 genes in the black modules (Figure 1(F); Table S2) were revealed as the hub genes and included for further analysis.

**Figure 2.**
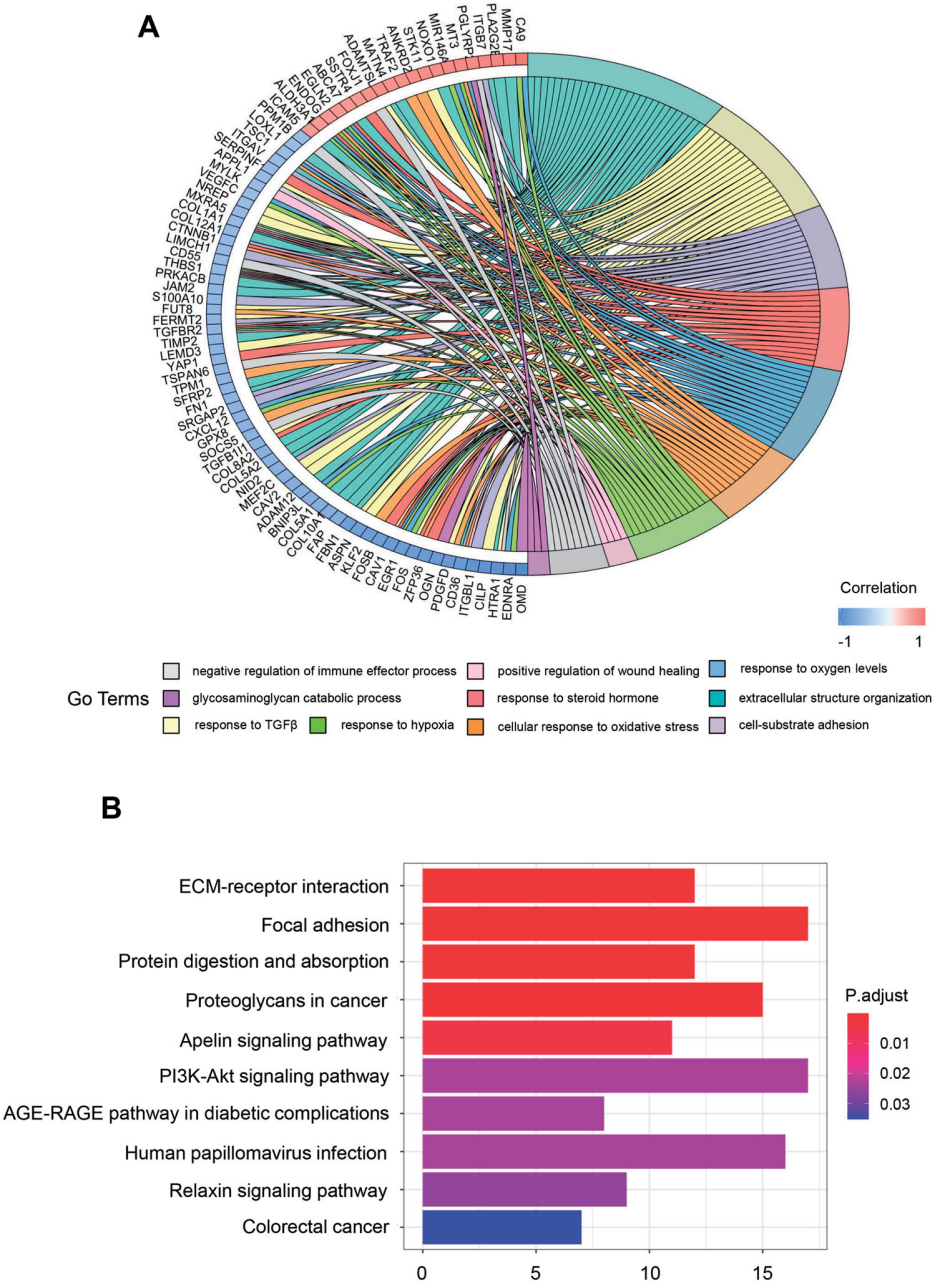
GO and KEGG enrichment analysis for metabolism-related hub genes. (A) Major GO enrichment terms in hub gene clusters correlated to metabolism; (B) KEGG pathway enrichment in hub gene clusters correlated to metabolism. GO: Gene ontology; KEGG: Kyoto Encyclopaedia of Genes and Genomes.

To better depict these hub genes' biologic activity, the GO and KEGG enrichment analysis were conducted. The GO analyses revealed that the dominant biological functions include extracellular structure organisation, cell-substrate adhesion, and positive regulation of wound healing. Meanwhile, these genes were involved in responses to transforming growth factor-beta and steroid hormone and the negative regulation of the immune effector process. Interestingly, these genes also play a role in the biological reaction towards different oxygen levels. Several genes were related to response to oxygen levels, cellular response to oxidative stress, and response hypoxia (Figure 2(A)). For KEGG analysis, pathways concerning extracellular matrix (ECM) regulation (ECM-receptor interaction, focal adhesion, and proteoglycans in cancer), nutrition regulation (protein digestion and absorption), and oncogenesis (PI3K-Akt signalling pathway) were closely related to the hub genes (Figure 2(B)). Surprisingly, the HPV (human papillomavirus) infection pathway is one of the most involved pathways. The HPV infection pathway is not only related to oncogenesis but also cancer-specific immune evasion. What’s more, the anti-tumour immune pathways can also be activated by antiviral signals [21].

We depicted PPI networks of hub genes *via* the online tool STRING (URL: https://string-db.org/) to achieve a clear vision of the interactions among hub genes (Figure S1). The threshold of interaction score for high confidence was set at 0.7, and disconnected nodes in the network were hidden. A total of 165 hub genes were involved in the construction of this regulatory network. Correspondent proteins of hub genes have a relatively high internal connections level, indicating a firm inter-module relationship. Notably, FN1, a gene involved in cell adhesion and migration processes, including embryogenesis, wound healing, blood coagulation, host defense, and metastasis, appears to be the centre of the PPI network. Previous studies suggested that FN1 is a major ECM component secreted by various epithelial or mesenchymal cell types in both physiologic and pathophysiologic situations in response to high glucose stimulations [22–24].

### Reveal of the overlap between TNBC specific immunity and glucose metabolism

**Figure 3.**
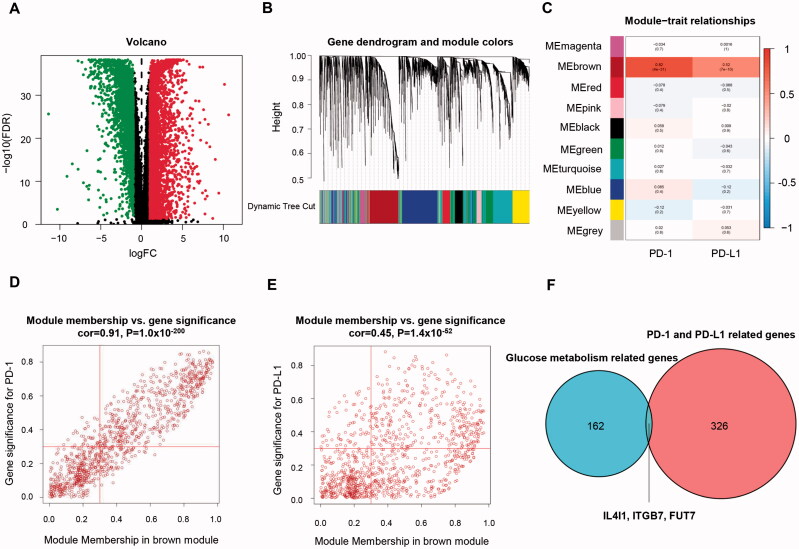
Identification of immune-related genes in TNBC. (A) Volcano plot showing differentially expressed genes. (B) Dendrogram plot with colour annotation. (C) Heatmap for the correlations of gene modules to PD-(L)1. Scatterplots for correlations between module membership and gene significance values concerning PD-1 (D) and PD-L1 (E) expression for the brown gene module. (F) Venn diagram of overlapping genes identified by the immune-related gene set and metabolic gene set. TNBC: Triple-negative breast cancer.

For the next step, we focus on revealing the overlap between the above genes and cancer-specific immunity. Genes related to PD-(L)1 expression was identified using the RNA profiles of 146 TNBC and 111 normal tissues retrieved from the TCGA database. 3288 upregulated and 2914 down-regulated differentially expressed genes are identified (Figure 3(A)). Ten major gene modules were established by further WGCNA (Figure 3(B)). Genes within the brown module have the strongest positive correlation with the increment of PD-(L)1 in BC (Figure 3(C)). Thus, 329 genes in the brown module with MM >0.3 and GS >0.3 were regarded as hub genes (Figure 3(D,E) and Table S3).

**Figure 4.**
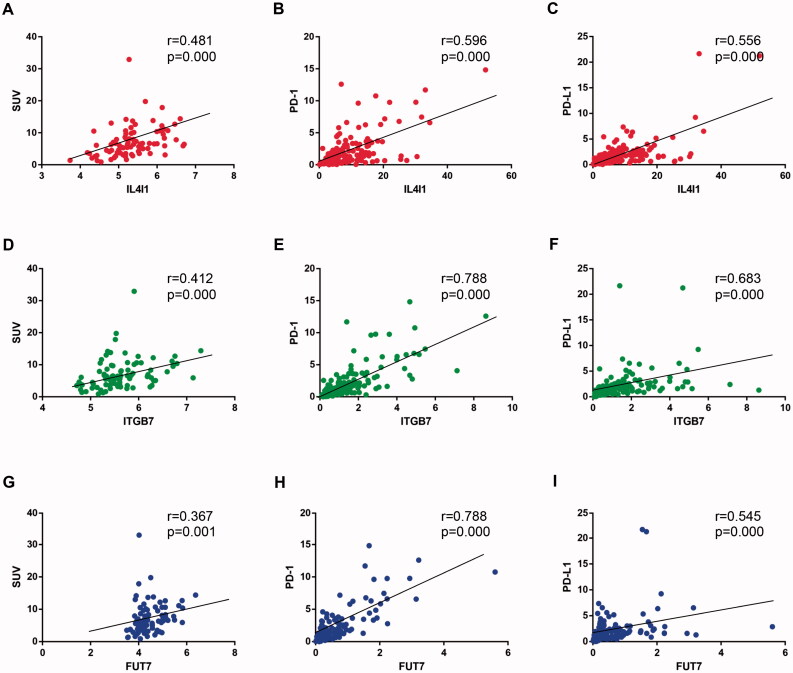
Correlation between hub gene expression and SUV and PD-(L)1 expression. The Spearman correlation coefficients between IL4I1 (A–C), ITGB7 (D to F), and FUT7 (G–I) expression and SUV, PD-1, and PD-L1 levels were shown. SUV: Standardised uptake value.

Most important, IL4I1, ITGB7, and FUT7 were revealed in both the cancer-immune gene set and glucose metabolic gene set (Figure 3(F)). They all had a moderate to strong positive relationship with the increased SUV and PD-(L)1 expression (Figure 4). These genes might be the potential linkage between cancer-specific immunity and glucose metabolism and were included for further analysis.

### The expression profile of IL4I1, ITGB7, and FUT7

**Figure 5.**
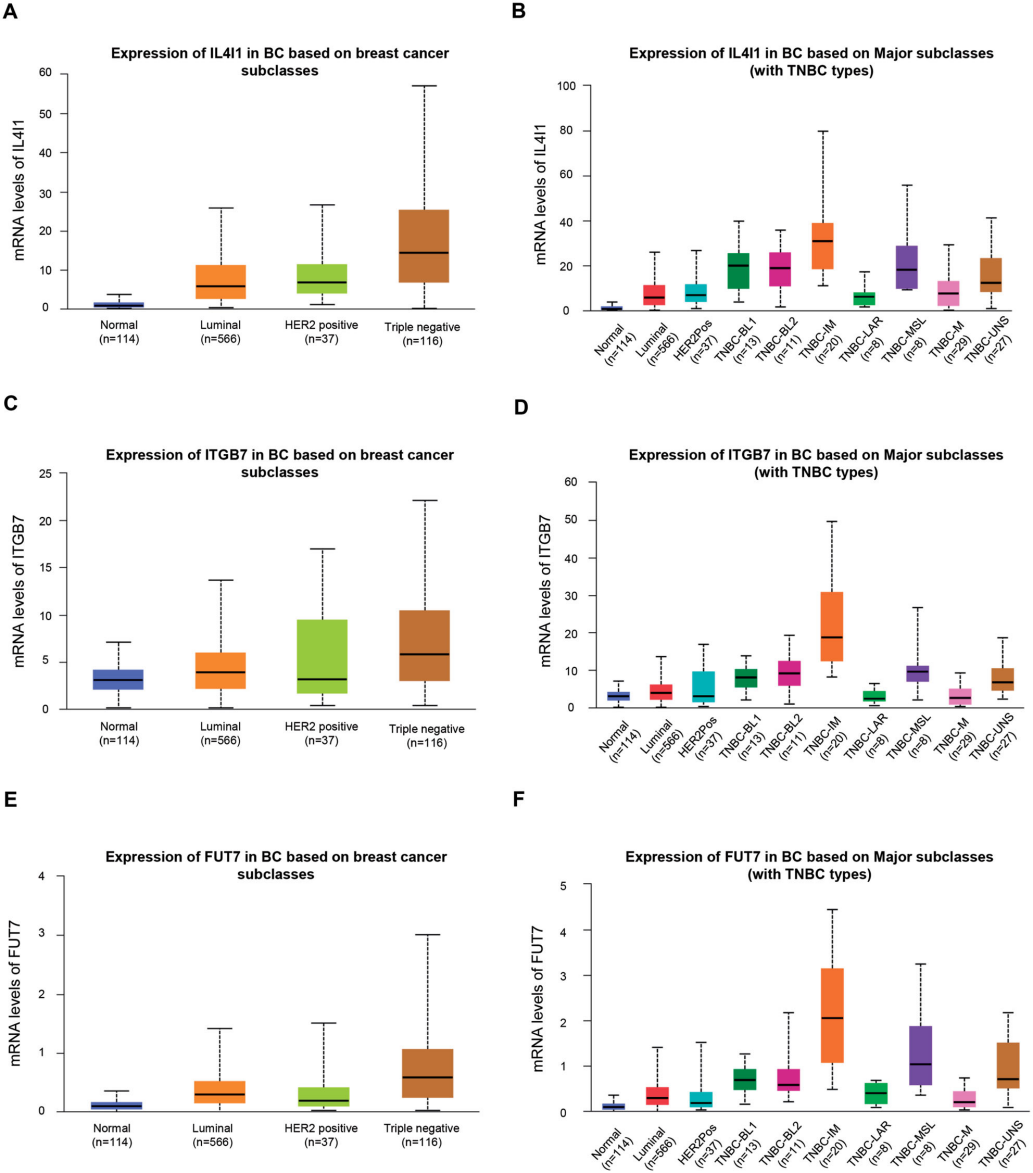
Expression levels of IL4I1 (A,B), ITGB7 (C,D), and FUT7 (E,F) in different breast cancer subtypes or TNBC subtypes. TNBC: triple-negative breast cancer.

Recent advances in genome-wide gene expression profiling analyses improved our understanding of TNBC and revealed at least six different molecular subtypes of TNBC, namely Basal-like 1 (BL1), basal-like 2 (BL2), immunomodulatory (IM), mesenchymal (M), mesenchymal stem-like (MSL) and luminal androgen receptor (LAR) [25]. We explored the mRNA expressions of hub genes in these molecular subtypes of TNBC using UALCAN (http://ualcan.path.uab.edu), which was an interactive online database to perform in-depth analyses of gene expression data from TCGA [26]. We found that all three genes were highly expressed in the IM subtype of TNBC (Figure 5). The IM subtype was characterised by significant gene enrichment in immune cell signalling, cytokine signalling, antigen processing and presentation, and signalling through core immune signal transduction pathways.

**Figure 6.**
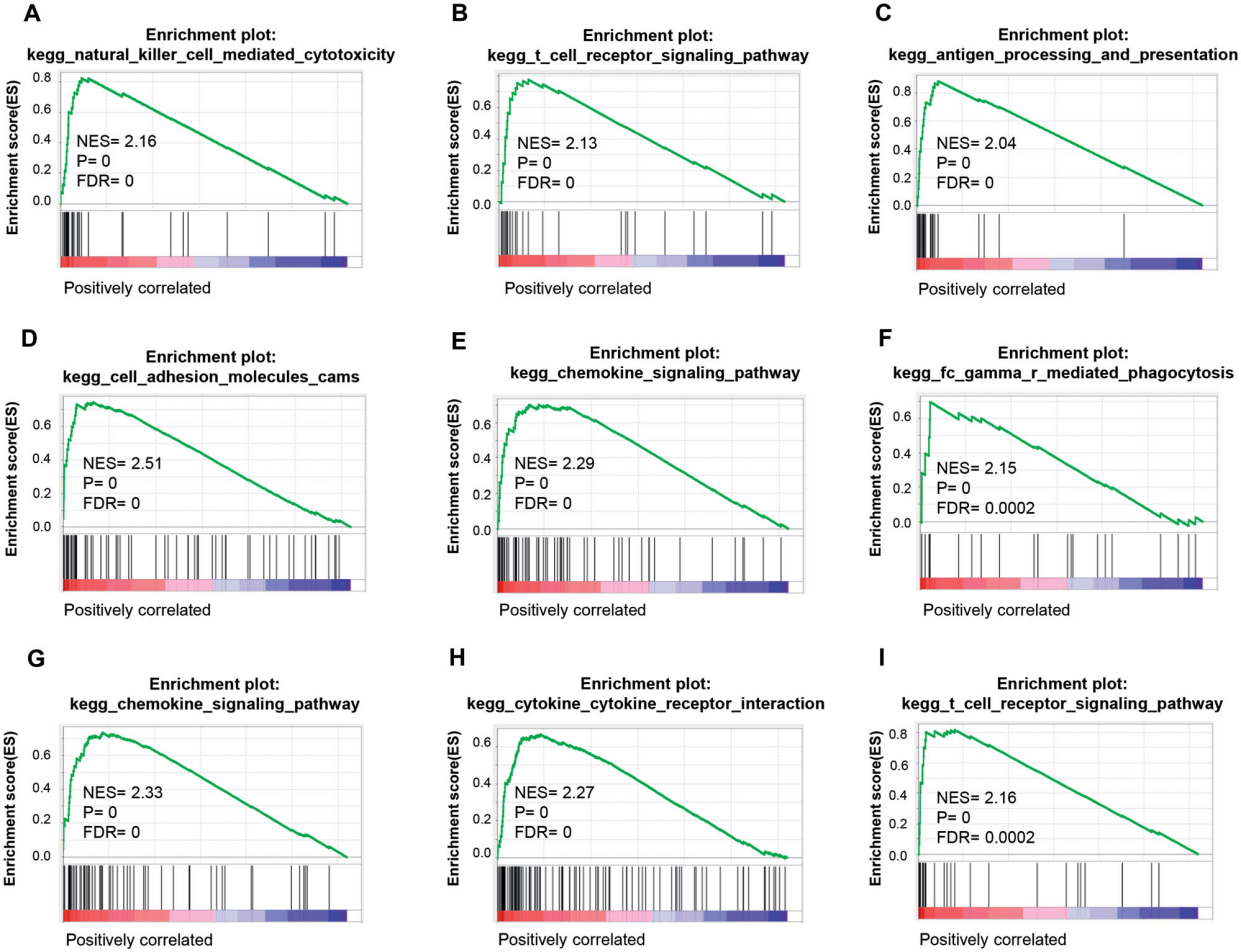
GSEA analysis of TNBC grouped by IL4I1, ITGB7, and FUT7 expression. GSEA was performed on RNA-Seq data of 146 TNBC samples grouped by IL4I1, ITGB7, and FUT7 expression levels, respectively. Representative pathways of IL4I1 (A–C), ITGB7 (D–F), and FUT7 (G–I) were shown. GSEA: gene set enrichment analysis.

Similar results were revealed by the single-gene GSEA analysis (Figure 6). A high expression of IL4L1 was related to pathways of NK cell-mediated cytotoxicity, T cell receptor signalling pathway, and antigen processing and presentation. The representative pathways associated with ITGB7, including cell adhesion molecules (CAMs), chemokine signalling pathway, and Fc gamma R mediated phagocytosis. FUT7 exhibited a potent relationship with the chemokine signalling pathway, cytokine cytokine-receptor interaction, and T cell receptor signalling pathway.

### Immune cell infiltration analysis

**Figure 7.**
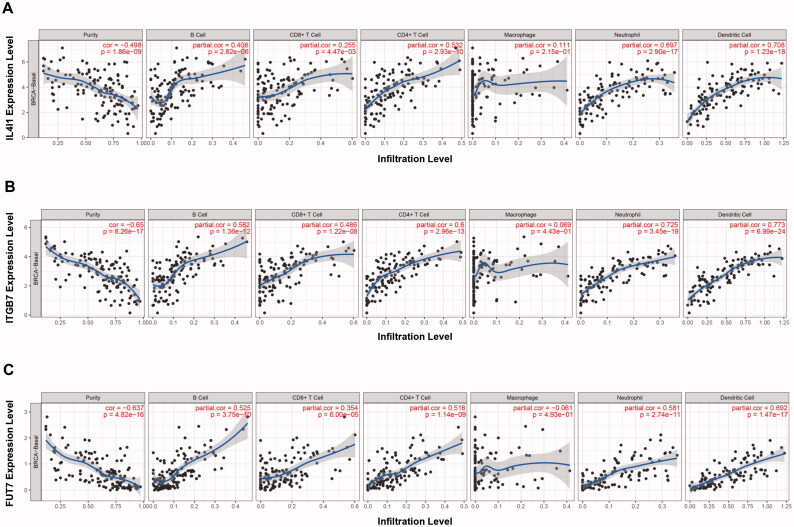
Immune cell infiltration analysis. The TIMER was used to evaluate the correlations between immune cell abundance and IL4I1 (A), ITGB7 (B), and FUT7 (C) expressions in basal breast cancer. TIMER: Tumour immune estimation resource.

The relationship of IL4I1, ITGB7, and FUT7 and immune cell infiltration characterisations were further explored using TIMER databases (Figure 7). The expression of IL4I1 correlated with the infiltrating levels of B cells, CD4+ T cells, neutrophils, and DC cells positively. Similarly, ITGB7 and FUT7 were both positively correlated with the infiltrating levels of B cells, CD8+ T cells, CD4+ T cells, neutrophils, and DC cells. Most importantly, analysis of TIMER and GEPIA databases revealed that IL4I1, ITGB7, and FUT7 all have potent positive associations with the abundance of Treg cells, indicated their immunosuppressive effects (Figure S2; Table 1). Similar immune cell infiltration characterisations were found in the other two types (Luminal and HER2 positive) breast cancer (Figure S3). Furthermore, we observed a significant positive correlation between the expression of three hub genes and the expression of exhausted T cell markers, including LAG-3, TIM-3, CTLA-4, and CXCL13. Meanwhile, the levels of IL4I1, ITGB7, and FUT7 expressions also correlate with the expression of marker genes for TAM, M2, Tfh, and Th1 cell infiltration (Table 1).

**Table 1.** Correlation analysis between IL4I1, ITGB7, and FUT7 and relate genes and markers of immune cells in GEPIA2.

Description	Gene markers	IL4I1		ITGB7		FUT7	
		Core	*p*	Core	*p*	Core	*p*
T cell (general)	CD3D	0.63	.000	0.73	.000	0.79	.000
	CD3E	0.60	.000	0.73	.000	0.76	.000
	CD2	0.64	.000	0.74	.000	0.75	.000
TAM	CCL2	0.55	.000	0.51	.000	0.49	.000
	CD68	0.67	.000	0.50	.000	0.46	.000
	IL10	0.59	.000	0.52	.000	0.44	.000
M1 macrophage	iNOS	–	–	–	–	–	–
	IRF5	0.41	.000	0.43	.000	0.43	.000
	COX2	0.13	.000	0.22	.000	0.20	.000
M2 macrophage	CD163	0.70	.000	0.56	.000	0.55	.000
	VSIG4	0.47	.000	0.39	.000	0.40	.000
	MS4A4A	0.58	.000	0.52	.000	0.50	.000
Tfh	BCL6	–	–	–	–	–	–
	IL21	0.49	.000	0.55	.000	0.49	.000
Th1	T-bet	0.63	.000	0.74	.000	0.76	.000
	STAT4	0.51	.000	0.64	.000	0.64	.000
	STAT1	0.55	.000	0.51	.000	0.38	.000
	IFN-γ	0.61	.000	0.65	.000	0.62	.000
	TNF-α	0.49	.000	0.41	.000	0.43	.000
Th2	GATA3	−0.38	.000	−0.29	.000	−0.29	.000
	STAT6	–	–	–	–	–	–
	STAT5A	0.21	.000	0.29	.000	0.32	.000
	IL13	0.29	.000	0.29	.000	0.28	.000
Th17	STAT3	–	–	–	–	–	–
	IL17A	0.27	.000	0.27	.000	0.25	.000
Treg	FOXP3	0.68	.000	0.65	.000	0.66	.000
	CCR8	0.59	.000	0.54	.000	0.50	.000
	TGFβ	0.30	.000	0.26	.000	0.41	.000
T cell exhaustion	CTLA4	0.72	.000	0.72	.000	0.70	.000
	LAG3	0.69	.000	0.66	.000	0.64	.000
	TIM-3	0.66	.000	0.50	.000	0.50	.000
	CXCL13	0.48	.000	0.50	.000	0.55	.000

TAM: tumour-associated macrophage; Th: T helper cell; Tfh: Follicular helper T cell; Treg: regulatory T cell; Cor: R value of Spearman’s correlation. “–” represents |R|<0.1.

### Prognosis analysis

We evaluate the prognosis value of IL4I1, ITGB7 and FUT7 in TNBC by using PrognoScan (Table S4). Results showed that all of them were potent negative prognostic factors for OS (Overall Survival) (HR: 1.49 [1.02–2.18], *p* = .041; 1.85 [1.23–2.78] *p* = .003; 2.07 [1.08–3.97] *p* = .028, for IL4I1, ITGB7, and FUT7, respectively). IL4I1 and ITGB7 are also negative predictors for relapse (HR: 1.86 [1.25–2.76], *p* = .002; 4.41 [1.07–18.25], *p* = .041 for IL4I1 and ITGB7, respectively). GSE1456, GSE9195, GSE9893, GSE9195 and GSE3143 were involved in above survival analysis.

## Discussion

This study provides novel insights into the linkage between two important hallmarks in cancer, glucose metabolism, and cancer-specific immunity. IL4I1, ITGB7, and FUT7, which were identified by comprehensive bioinformatic analysis, may play a vital role in the tumour immune microenvironment and hold the potential of enhancing ICB treatment by remodelling glucose metabolism. This hypothesis was also confirmed by gene enrichment analysis.

In this study, the glucose uptake intensities were presented through SUV assessed by FDG-PET. FDG-PET is a well-recognized imaging tool in reflecting glucose metabolism, and SUV is an objective indicator of glucose uptake [27]. Compared with normal tissue, tumours often demonstrate an increased FDG-PET signal, which reflects a high rate of radiotracer uptake through membrane glucose transporters, phosphorylation by one of several hexokinase enzymes, and the resultant intracellular trapping of the radiotracer, which is not further metabolised in the cell [28,29]. Indeed, there are concerns since the genomic characteristics of SUV have not been fully explored, and the validity of using SUV to reflect the complex effects of glucose metabolism on cancer progression is questionable and inaccurate. However, to date, FDG-PET is the only reflection of cancer metabolic behaviour actually occurring in the human body [30]. Meanwhile, plenty of previous studies have proved that SUV evaluated by FDG-PET is highly clinically and pathologically relevant [31,32].

Moreover, the expression level of PD-(L)1 is used as a biomarker for cancer-specific immunity in our study, in accordance with previous studies, since they were the most important immune checkpoints and well-known therapeutic targets [33–35]. We believe that genes related to PD-(L)1 hold the most substantial potential for future clinical application.

IL4I1, ITGB7, and FUT7 were identified in this study as key genes. In previous studies, they were considered to be important modifiers in tumour immune microenvironment and metabolism. IL4I1 is a bona fide immunosuppressive enzyme participating in the escape of malignancies *in vivo* [36]. It accelerates tumour growth by limiting the CD8 T cell-mediated immune response in a mouse model of melanoma cell transplantation [37,38]. The immune regulatory functions of IL4I1 have been attributed to either the depletion of amino acids, the promotion of Treg differentiation, or the upregulation of PD-1 [39,40]. What’s more, a recent study demonstrated that IL4I1 is a major aryl hydrocarbon receptor (AHR)-activating enzyme and endowed cancer cells with the capacity of migration and metastasis [41]. ITGB7 is a key factor in the ITGB7/C/EBPβ signalling axis and integrin β4/FAK/SOX2/HIF-1α signalling pathway, which plays a vital part in glycolysis-induced cell growth and proliferation [42,43]. The high involvement of C/EBPβ signalling and HIF-1α signalling pathway with immune inhabitation was also well-established [44]. Meanwhile, Fucosyltransferase VII (FUT7) is one of the a1,3-fucosyltransferases family that catalyses the final fucosylation step in generating a unique glycosylated product sialyl Lewis X (sLeX) [45,46]. sLeX can serve as ligands for E- or P-selectin expressed on the cell surface and results in cancer metastasis and angiogenesis [11,46]. Additionally, increased fucosylated activity was also reported positively relative to increased glucose uptake by the pancreatic, lung, and ovarian carcinoma cell lines [47]. According to previous studies, the upregulation of IL4I1 maybe a result of naturally occurring Single Nucleotide Polymorphisms (SNPs) [48], the DNA demethylation of FUT7 could lead to its overexpression [49], and highly expressed ITGB7 may be induced by TGF-beta1 [50,51]. However, the potential basis for upregulation of above genes remains unknown in patients with breast cancers.

One of the most interesting findings observed in this study is the positive relationship between selected genes and the abundance of T cells, especially for CD4+ T cells and Treg cells. The mechanism of this connection is unclear. We assume that increased Treg cell infiltration may be the reason for inadequate responses to ICB treatment in TNBC, since Treg cells display functional instability and heterogeneity, and increased Treg frequency is often associated with reduced activity of antitumor cytotoxic T lymphocytes and worse prognosis [52]. The strong correlation between the expression of hub genes identified and the markers of T cell exhaustion may also exacerbate this situation. However, this hypothesis should be investigated in future studies.

There are still some limitations in this study. The major limitation of this study is that only *in silico* analysis were conducted. Therefore, all study results should be confirmed by *in vivo* or *in vitro* experiments in the future. Second, this study only applied analysis on the mRNA expression level. Thus, multi-omics analyses are warranted in the future.

In conclusion, IL4I1, ITGB7, and FUT7 were identified as the hub genes between two hallmarks in cancer, glucose metabolism, and cancer-specific immunity *via* comprehensive bioinformatic analysis. The GO and pathway enrichment analysis suggest that they may be potential targets for enhancing ICB treatment by reprogramming the tumour microenvironment and remodelling tumour metabolism.

## Supplementary Material

Supplemental Material

## Data Availability

The data that support the findings of this study are openly available in Gene Expression Omnibus (GEO) database, GSE135565 dataset (http://www.ncbi.nlm.nih.gov/geo/).

## References

[1] Bianchini G, Balko JM, Mayer IA, et al. Triple-negative breast cancer: challenges and opportunities of a heterogeneous disease. Nat Rev Clin Oncol. 2016;13(11):674–690. 27184417 10.1038/nrclinonc.2016.66 PMC5461122

[2] Foulkes WD, Smith IE, Reis-Filho JS. Triple-negative breast cancer. N Engl J Med. 2010;363(20):1938–1948. 21067385 10.1056/NEJMra1001389

[3] Pardoll DM. The blockade of immune checkpoints in cancer immunotherapy. Nat Rev Cancer. 2012;12(4):252–264. 22437870 10.1038/nrc3239 PMC4856023

[4] Adams S, Gatti-Mays ME, Kalinsky K, et al. Current landscape of immunotherapy in breast cancer: a review. JAMA Oncol. 2019;5(8):1205–1214. 30973611 10.1001/jamaoncol.2018.7147 PMC8452050

[5] Vranic S, Cyprian FS, Gatalica Z, et al. PD-L1 status in breast cancer: current view and perspectives. Semin Cancer Biol. 2021;72:146–154. 31883913 10.1016/j.semcancer.2019.12.003

[6] Chang C-H, Qiu J, O’Sullivan D, et al. Metabolic competition in the tumor microenvironment is a driver of cancer progression. Cell. 2015;162(6):1229–1241. 26321679 10.1016/j.cell.2015.08.016 PMC4864363

[7] Brand A, Singer K, Koehl GE, et al. LDHA-associated lactic acid production blunts tumor immunosurveillance by T and NK cells. Cell Metab. 2016;24(5):657–671. 27641098 10.1016/j.cmet.2016.08.011

[8] Boussiotis VA. Molecular and biochemical aspects of the PD-1 checkpoint pathway. N Engl J Med. 2016;375(18):1767–1778. 27806234 10.1056/NEJMra1514296 PMC5575761

[9] Li CW, Lim SO, Chung EM, et al. Eradication of triple-negative breast cancer cells by targeting glycosylated PD-L1. Cancer Cell. 2018;33(2):187–201 e10. 29438695 10.1016/j.ccell.2018.01.009 PMC5824730

[10] Li CW, Lim SO, Xia W, et al. Glycosylation and stabilization of programmed death ligand-1 suppresses T-cell activity. Nat Commun. 2016;7:12632. 27572267 10.1038/ncomms12632 PMC5013604

[11] Sun L, Li CW, Chung EM, et al. Targeting glycosylated PD-1 induces potent antitumor immunity. Cancer Res. 2020;80(11):2298–2310. 32156778 10.1158/0008-5472.CAN-19-3133 PMC7272274

[12] Martinez-Outschoorn UE, Peiris-Pagés M, Pestell RG, Sotgia F, et al. Cancer metabolism: a therapeutic perspective. Nat Rev Clin Oncol . 2017;14(1):11–31. 27141887 10.1038/nrclinonc.2016.60

[13] Gong Y, Ji P, Yang Y-S, et al. Metabolic-pathway-based subtyping of triple-negative breast cancer reveals potential therapeutic targets. Cell Metab. 2021;33(1):51–64.e9. 33181091 10.1016/j.cmet.2020.10.012

[14] Kim SK, Ahn SG, Mun JY, et al. Genomic signature of the standardized uptake value in (18)F-fluorodeoxyglucose positron emission tomography in breast cancer. Cancers. 2020;12(2):497. 32093417 10.3390/cancers12020497 PMC7072341

[15] Kang S, Kim EH, Hwang JE, et al. Prognostic significance of high metabolic activity in breast cancer: PET signature in breast cancer. Biochem Biophys Res Commun. 2019;511(1):185–191. 30777332 10.1016/j.bbrc.2019.02.035 PMC8356554

[16] Langfelder P, Horvath S. WGCNA: an R package for weighted correlation network analysis. BMC Bioinf . 2008;9:559. 10.1186/1471-2105-9-559 PMC2631488 19114008

[17] Li T, Wernersson R, Hansen RB, et al. A scored human protein-protein interaction network to catalyze genomic interpretation. Nat Methods. 2017;14(1):61–64. 27892958 10.1038/nmeth.4083 PMC5839635

[18] Mizuno H, Kitada K, Nakai K, et al. PrognoScan: a new database for meta-analysis of the prognostic value of genes. BMC Med Genomics. 2009;2:18. 19393097 10.1186/1755-8794-2-18 PMC2689870

[19] Li T, Fan J, Wang B, et al. TIMER: a web server for comprehensive analysis of tumor-infiltrating immune cells. Cancer Res. 2017;77(21):e108-e10. 29092952 10.1158/0008-5472.CAN-17-0307 PMC6042652

[20] Gu Y, Li X, Bi Y, et al. CCL14 is a prognostic biomarker and correlates with immune infiltrates in hepatocellular carcinoma. Aging. 2020;12(1):784–807. 31927532 10.18632/aging.102656 PMC6977663

[21] Bowling EA, Wang JH, Gong F, et al. Spliceosome-targeted therapies trigger an antiviral immune response in triple-negative breast cancer. Cell. 2021;184(2):384–403.e21. 33450205 10.1016/j.cell.2020.12.031 PMC8635244

[22] Saik OV, Klimontov VV. Bioinformatic reconstruction and analysis of gene networks related to glucose variability in diabetes and its complications. Int J Mol Sci. 2020;21(22):8691. 33217980 10.3390/ijms21228691 PMC7698756

[23] Zhang H, Wang JW, Xu Y, et al. Effect of β-(3,4-dihydroxyphenyl)lactic acid on oxidative stress stimulated by high glucose levels in human peritoneal mesothelial cells. J Int Med Res. 2012;40(3):943–953. 22906267 10.1177/147323001204000313

[24] Alvarez ML, DiStefano JK. Functional characterization of the plasmacytoma variant translocation 1 gene (PVT1) in diabetic nephropathy. PLoS One. 2011;6(4):e18671. 21526116 10.1371/journal.pone.0018671 PMC3081298

[25] Lehmann BD, Bauer JA, Chen X, et al. Identification of human triple-negative breast cancer subtypes and preclinical models for selection of targeted therapies. J Clin Invest. 2011;121(7):2750–2767. 21633166 10.1172/JCI45014 PMC3127435

[26] Chandrashekar DS, Bashel B, Balasubramanya SAH, et al. UALCAN: a portal for facilitating tumor subgroup gene expression and survival analyses. Neoplasia. 2017;19(8):649–658. 28732212 10.1016/j.neo.2017.05.002 PMC5516091

[27] Ahn SG, Park JT, Lee HM, et al. Standardized uptake value of 18F-fluorodeoxyglucose positron emission tomography for prediction of tumor recurrence in breast cancer beyond tumor burden. Breast Cancer Res. 2014;16(6):502. 25551703 10.1186/s13058-014-0502-y PMC4308858

[28] Jadvar H, Alavi A, Gambhir SS. 18F-FDG uptake in lung, breast, and colon cancers: molecular biology correlates and disease characterization. J Nucl Med. 2009;50(11):1820–1827. 19837767 10.2967/jnumed.108.054098 PMC2783751

[29] Palaskas N, Larson SM, Schultz N, et al. 18F-fluorodeoxy-glucose positron emission tomography marks MYC-overexpressing human basal-like breast cancers. Cancer Res. 2011;71(15):5164–5174. 21646475 10.1158/0008-5472.CAN-10-4633 PMC3148325

[30] Fletcher JW, Djulbegovic B, Soares HP, et al. Recommendations on the use of 18F-FDG PET in oncology. J Nucl Med. 2008;49(3):480–508. 18287273 10.2967/jnumed.107.047787

[31] Shimoda W, Hayashi M, Murakami K, et al. The relationship between FDG uptake in PET scans and biological behavior in breast cancer. Breast Cancer. 2007;14(3):260–268. 17690502 10.2325/jbcs.14.260

[32] Ahn SG, Lee J-H, Lee HW, et al. Comparison of standardized uptake value of 18F-FDG-PET-CT with 21-gene recurrence score in estrogen receptor-positive, HER2-negative breast cancer. PLoS One. 2017;12(4):e0175048. 28419166 10.1371/journal.pone.0175048 PMC5395149

[33] Xu T, Wang Z, Dong M, et al. Chloride intracellular channel protein 2: prognostic marker and correlation with PD-1/PD-L1 in breast cancer. Aging. 2020;12(17):17305–17327. 32915772 10.18632/aging.103712 PMC7521498

[34] Tomioka N, Azuma M, Ikarashi M, et al. The therapeutic candidate for immune checkpoint inhibitors elucidated by the status of tumor-infiltrating lymphocytes (TILs) and programmed death ligand 1 (PD-L1) expression in triple negative breast cancer (TNBC). Breast Cancer. 2018;25(1):34–42. 28488168 10.1007/s12282-017-0781-0

[35] Park IH, Kong SY, Ro JY, et al. Prognostic implications of tumor-infiltrating lymphocytes in association with programmed death ligand 1 expression in early-stage breast cancer. Clin Breast Cancer. 2016;16(1):51–58. 26364145 10.1016/j.clbc.2015.07.006

[36] Lasoudris F, Cousin C, Prevost-Blondel A, et al. IL4I1: an inhibitor of the CD8+ antitumor T-cell response in vivo. Eur J Immunol. 2011;41(6):1629–1638. 21469114 10.1002/eji.201041119 PMC3472400

[37] Bod L, Lengagne R, Wrobel L, et al. IL4-induced gene 1 promotes tumor growth by shaping the immune microenvironment in melanoma. Oncoimmunology. 2017;6(3):e1278331. 28405502 10.1080/2162402X.2016.1278331 PMC5384381

[38] Yue Y, Huang W, Liang J, et al. IL4I1 is a novel regulator of M2 macrophage polarization that Can inhibit T cell activation via L-tryptophan and arginine depletion and IL-10 production. PLoS One. 2015;10(11):e0142979. 26599209 10.1371/journal.pone.0142979 PMC4658051

[39] Molinier-Frenkel V, Prévost-Blondel A, Castellano F. The IL4I1 enzyme: a new player in the immunosuppressive tumor microenvironment. Cells. 2019;8(7):757. 31330829 10.3390/cells8070757 PMC6678094

[40] Mezrich JD, Fechner JH, Zhang X, et al. An interaction between kynurenine and the aryl hydrocarbon receptor can generate regulatory T cells. J Immunol. 2010;185(6):3190–3198. 20720200 10.4049/jimmunol.0903670 PMC2952546

[41] Sadik A, Somarribas Patterson LF, Ozturk S, et al. IL4I1 is a metabolic immune checkpoint that activates the AHR and promotes tumor progression. Cell. 2020;182(5):1252–1270.e34. 32818467 10.1016/j.cell.2020.07.038

[42] Chai Z, Yang Y, Gu Z, et al. Recombinant viral capsid protein L2 (rVL2) of HPV 16 suppresses cell proliferation and glucose metabolism via ITGB7/C/EBPbeta signaling pathway in cervical cancer cell lines. Onco Targets Ther. 2019;12:10415–10425. 31819523 10.2147/OTT.S228631 PMC6890187

[43] Gan L, Meng J, Xu M, et al. Extracellular matrix protein 1 promotes cell metastasis and glucose metabolism by inducing integrin beta4/FAK/SOX2/HIF-1alpha signaling pathway in gastric cancer. Oncogene. 2018;37(6):744–755. 29059156 10.1038/onc.2017.363

[44] Cha JH, Chan LC, Li CW, et al. Mechanisms controlling PD-L1 expression in cancer. Mol Cell. 2019;76(3):359–370. 31668929 10.1016/j.molcel.2019.09.030 PMC6981282

[45] Liu S, Zhang Y, Liu Y, et al. FUT7 antisense sequence inhibits the expression of FUT7/sLeX and adhesion between embryonic and uterine cells. IUBMB Life. 2008;60(7):461–466. 18553500 10.1002/iub.62

[46] Liang JX, Gao W, Cai L. Fucosyltransferase VII promotes proliferation via the EGFR/AKT/mTOR pathway in A549 cells. Onco Targets Ther. 2017;10:3971–3978. 28860805 10.2147/OTT.S140940 PMC5558582

[47] Babiuch K, Dag A, Zhao J, et al. Carbohydrate-specific uptake of fucosylated polymeric micelles by different cancer cell lines. Biomacromolecules. 2015;16(7):1948–1957. 26057004 10.1021/acs.biomac.5b00299

[48] Molinier-Frenkel V, Mestivier D, Castellano F. Alterations of the immunosuppressive IL4I1 enzyme activity induced by naturally occurring SNP/mutations. Genes Immun. 2016;17(2):148–152. 26673964 10.1038/gene.2015.55

[49] Pink M, Ratsch BA, Mardahl M, et al. Imprinting of skin/inflammation homing in CD4+ T cells is controlled by DNA methylation within the fucosyltransferase 7 gene. J Immunol. 2016;197(8):3406–3414. 27591321 10.4049/jimmunol.1502434

[50] Wang J, Xiang H, Lu Y, et al. Role and clinical significance of TGF-β1 and TGF-βR1 in malignant tumors (Review). Int J Mol Med. 2021;47(4):4888. 10.3892/ijmm.2021.4888 PMC7895515 33604683

[51] Lim SP, Leung E, Krissansen GW. The beta7 integrin gene (Itgb-7) promoter is responsive to TGF-beta1: defining control regions. Immunogenetics. 1998;48(3):184–195. 9683663 10.1007/s002510050422

[52] Togashi Y, Shitara K, Nishikawa H. Regulatory T cells in cancer immunosuppression – implications for anticancer therapy. Nat Rev Clin Oncol. 2019;16(6):356–371. 30705439 10.1038/s41571-019-0175-7

