# Supplementary figures and images for: Integrated analysis reveals the participation of IL4I1, ITGB7, and FUT7 in reshaping the TNBC immune microenvironment by targeting glycolysis
Source: Ann Med. 2021 Jun 16;53(1):916–28. doi: 10.1080/07853890.2021.1937694 (PMC8604452; doi:10.1080/07853890.2021.1937694)

A

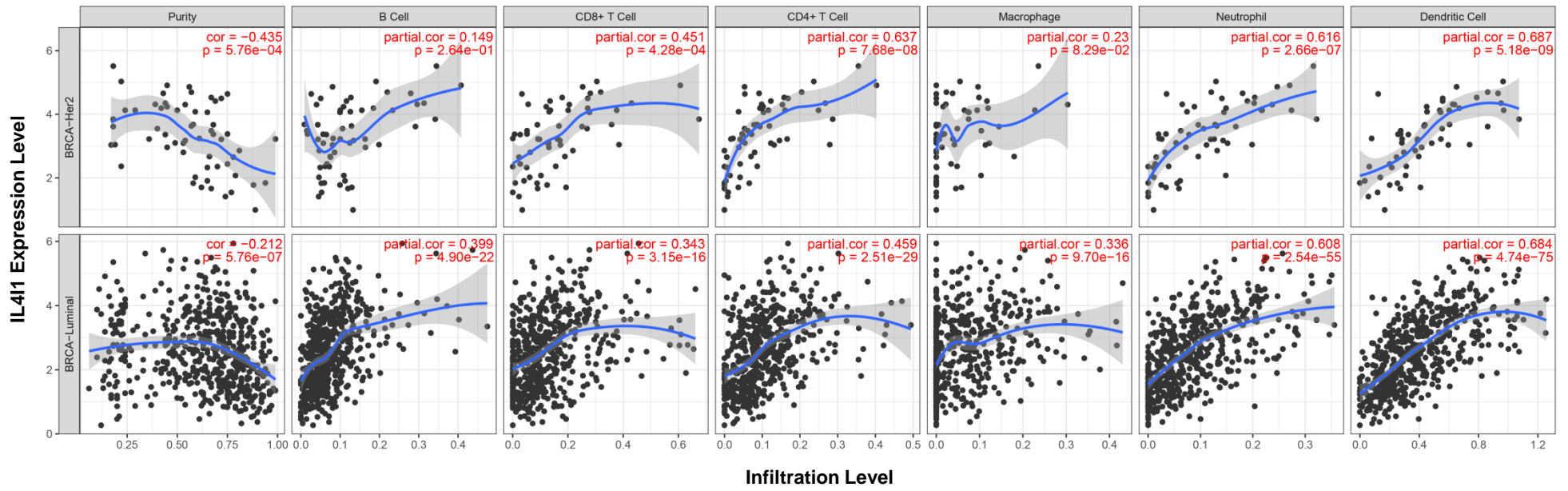

B

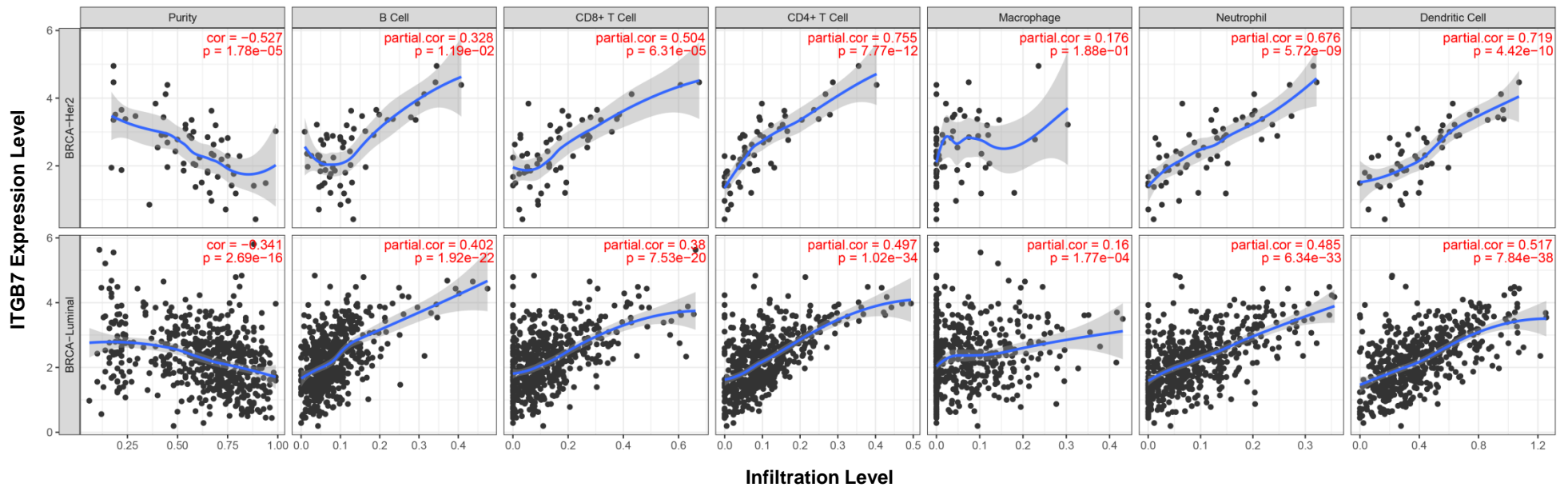

C

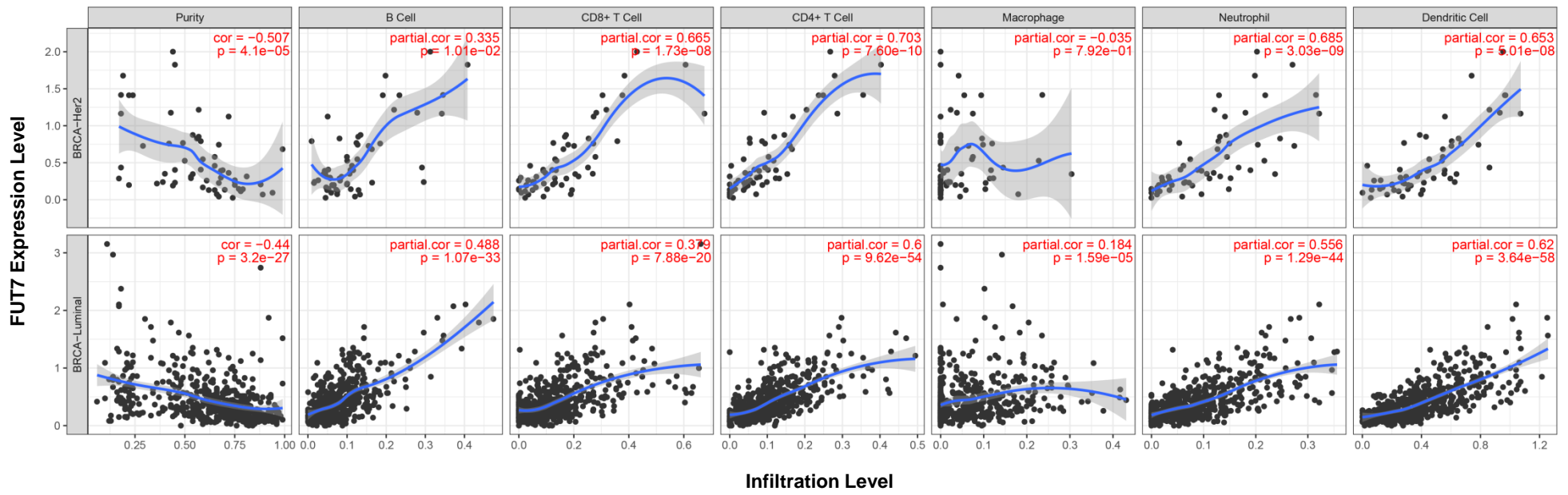

Supplement: Supplemental Material [file IANN_A_1937694_SM2063.zip › Revised_Figure_S3._Immune_cell_infiltration_analysis.pdf]
