# Supplementary material for: Integrated analysis reveals the participation of IL4I1, ITGB7, and FUT7 in reshaping the TNBC immune microenvironment by targeting glycolysis
Source: Ann Med. 2021 Jun 16;53(1):916–28. doi: 10.1080/07853890.2021.1937694 (PMC8604452; doi:10.1080/07853890.2021.1937694)
Supplement: Supplemental Material [file IANN_A_1937694_SM2063.zip › Fig_S3_Revised_Manuscript_Target_Metabolism_to_Enhance_ICB_Efficacy_in_TNBC.doc]

**Integrated analysis reveals the participation of IL4I1, ITGB7, and FUT7 in reshaping the TNBC immune microenvironment by targeting glycolysis**

Tao Xu^1, 2, 3#^, Jiahao Liu^1, 3#^, Yu Xia^1, 3#^, Zhi Wang^1,3^, Xingrui Li^2*^, Qinglei Gao^1,3*^

1 Cancer Biology Research Center (Key Laboratory of the Ministry of Education), Tongji Hospital, Tongji Medical College, Huazhong University of Science and Technology, Wuhan, China;

2 Department of Thyroid and Breast Surgery, Tongji Hospital, Tongji Medical College, Huazhong University of Science and Technology, Wuhan, China.

3 Department of Obstetrics and Gynecology, Tongji Hospital, Tongji Medical College, Huazhong University of Science and Technology, Wuhan, China

\# These authors contributed equally as senior authors.

*** Correspondence:**

Prof. Qinglei Gao, Cancer Biology Research Center, Key laboratory of the ministry of education, Tongji Hospital, Tongji Medical College, Huazhong University of Science and Technology, 1095 Jiefang Ave, Wuhan 430000, China. E-mail: qingleigao@hotmail.com;

Prof. Xingrui Li, Department of Thyroid and Breast Surgery, Tongji Hospital, Tongji Medical College, Huazhong University of Science and Technology, 1095 Jiefang Ave, Wuhan 430000, China. E-mail: lixingrui@tjh.tjmu.edu.cn.

**Short Title: Target Metabolism to Enhance ICB Efficacy in TNBC**

**Abstract**

**BACKGROUND**: Despite exceptional progress in cancer immunotherapy, the overall response rate of immune checkpoint blockade (ICB) in triple-negative breast cancer (TNBC) patients remains unsatisfactory. Meanwhile, accumulating evidence indicated that glucose metabolic reprogramming could modulate immunotherapy efficacy. However, transcriptomic linkages between glucose metabolism and immune microenvironments remain poorly investigated.

**METHODS**: Genes' relationship with glucose metabolism and TNBC-specific immune was demonstrated by weighted gene co-expression network analysis (WGCNA). The glucose metabolic capability was estimated by standardized uptake value (SUV), an indicator of glucose uptake in 18F-fluorodeoxyglucose positron emission tomography (FDG-PET), and a reflection of cancer metabolic behavior actually occurring in the human body. PD-(L)1 expression was used to reflect the efficacy of immunotherapy. Additionally, immune infiltration, survival, and gene co-expression profiles were provided to understand target genes better.

**RESULTS**: Comprehensive analysis revealing that IL4I1, ITGB7, and FUT7 hold the potential to reinforce immunotherapy by reshaping glucose metabolism in TNBC. These results were verified by functional enrichment analysis, which demonstrated that IL4I1, ITGB7, and FUT7 were correlated with immune-related signaling pathways and extracellular microenvironment reprogramming. The expressions of IL4I1, ITGB7, and FUT7 showed potent positive correlations with increased Treg and Macrophage cell infiltration and exhausted T cell markers, including LAG-3, TIM-3, CTLA-4, and CXCL13, suggesting their roles in altering tumor immune microenvironment. Meanwhile, the overexpression of these genes was also related to poor prognosis.

**CONCLUSION**: IL4I1, ITGB7, and FUT7 may be the hub genes that link two hallmarks in cancer, glucose metabolism, and cancer-specific immunity. They may be potential targets for enhancing ICB treatment by reprogramming the tumor microenvironment and remodeling tumor metabolism.

**Keywords:** triple-negative breast cancer; immunotherapy; glucose metabolism; FDG-PET; PD-(L)1

**Introduction**

Triple-negative breast cancer (TNBC) refers to breast cancers not expressing the estrogen receptor (ER), the progesterone receptor (PR), or human epidermal growth factor receptor 2 (HER2).^1^ Representing for about 15% of all breast cancer cases, TNBC is associated with a dreadful prognosis and a substantial unmet medical need.^2^ In the past few years, immune checkpoint blockers (ICB) led to a significant paradigm shift in oncology treatment, which may also offer promise of clinical benefits for TNBC.^3^ However, durable responses to immunotherapy were only observed in a small subset of patients.^4^ Thus, unlocking the therapeutic potential of ICB and expanding its role in TNBC treatment is of utmost clinical importance.

The treatment efficacy of ICB is largely dependent on the tumor microenvironment and immune regulators.^3^ Simultaneously, accumulating evidence showed that glucose metabolism contributes to both factors mentioned above substantially.^5^ In the tumor immune microenvironment, accelerated glucose catabolism plays a critical role in suppressing anti-tumor T cell functions by causing glucose deprivation.^6^ Moreover, increased glucose consumption and lactate production also lead to defective tumor immunosurveillance.^7^ With regard to immune modulate effects, previous studies shed light on the elaborate connection between glucose metabolism and PD-(L)1 (programmed death receptor-1, programmed death receptor ligand-1) expression.^8^ On the one hand, elevated glycolytic intensity leads to PD-L1 overexpression via PI3K–Akt–mTOR pathway activation, resulting in immune evasion and cancer progression.^6^ On the other hand, glucose metabolic deterioration potentially partakes in altered glycosylation of PD-(L)1, which is critical for maintaining its protein stability and cell surface localization.^9-11^ Meanwhile, the capacity of glucose uptake is a crucial hallmark for cancer itself.^12^ In 2020, Yue Gong and his college profiled the metabolic dysregulation in TNBC and divided TNBC into metabolic-pathway-based subtypes (MPS).^13^ According to their research, the MPS2 subtype is characterized by upregulated carbohydrate metabolism and exhibited the worst prognosis. Additionally, the standardized uptake value (SUV), as an indicator of glucose uptake in 18F-fluorodeoxyglucose positron emission tomography (FDG-PET), is closely related to the malignant phenotype of breast cancers.^14,15^

Despite the advances mentioned above, our knowledge of interactions between anti-tumor immunity and glucose metabolism remains relatively limited. In this study, insights connecting these two processes are revealed by conducting comprehensive bioinformatic analysis in TNBC. Genes identified in this study could be the nodal hub of the immunometabolism network and future target to reinforce ICB treatment efficacy.

**Methods**

**Data collection**

The mRNA expression data and SUV assessed by FDG-PET in 84 TNBC patients were downloaded from Gene Expression Omnibus (GEO) database, GSE135565 dataset (http://www.ncbi.nlm.nih.gov/geo/).^14^ RNA-Seq information for 146 TNBC and 111 normal tissues were downloaded from NCI's Genomic Data Commons the Cancer Genome Atlas cohort (TCGA) (https://gdc.cancer.gov/).

**Gene co-expression network construction**

The intensity of glucose uptake was measured by SUV, and the expression of PD-(L)1 was considered as a signature of the cancer-associated immunity. WGCNA was performed to identify gene modules correlated with a high level of SUV and PD-(L)1 expression.^16^ In this study, WGCNA was performed using R software (version 3.6.2). In the WGCNA algorithm, gene significance (GS, defined as the correlation between the gene and the standardized uptake value) and module membership (MM, defined as the correlation between the gene and its module) were used to quantify the configurations of modules and features.

**Functional enrichment and protein-protein interaction network Analysis**

The GO (Gene Ontology) annotation and KEGG (Kyoto Encyclopedia of Genes and Genomes) pathway enrichment analysis for targeted genes were accomplished by the R packages “clusterProfiler” and “enrichplot”. GSEA software was used to examine the different pathways related to target genes. STRING database was used to describe the PPI (protein-protein interaction) network.^17^

**Prognosis Analysis**

PrognoScan allows conducting systematic meta-analysis for prognosis on several GEO datasets. It is capable of evaluating the relationship between gene expression levels and various endpoints, including disease-specific survival (DSS), relapse-free survival (RFS), and overall survival (OS).^18^

**Immune Cell Infiltration Analysis**

The infiltration of immune cells, including CD4+ T cells, CD8+ T cells, B cells, neutrophils, macrophages, and dendritic cells, was estimated using the TIMER (Tumor IMmune Estimation Resource) algorithm, basing on RNA-Seq expression profile data of identical immune markers.^19^ The correlation between hub genes and immune cells abundance was calculated using Spearman's correlation analysis with correlation coefficient >0.3 indicates a positive/negative correlation. The Gene Expression Profiling Interactive Analysis (GEPIA) database (http://gepia.cancer-pku.cn) was used to analyze the relationship between the expression of hub genes and tumor-infiltrating immune cells marker genes [T cells, TAMs, M1 macrophages, M2 macrophages, T-helper (Th) cells, T-helper 17 (Th17) cells, follicular helper T (Tfh) cells, exhausted T cells, and Tregs], which were identified according to previous studies ^20^.

**Statistical analysis**

Differential analysis of RNA expression profile was performed with R (version 3.6.2). All data were standardized and analyzed using the “limma” package. Differential genes were screened according to (corrected p < 0.05, |logFC| >1). Data obtained from TIMER and PrognoScan were presented as HR and *P* values or Cox P values upon the log-rank test. *P* < 0.05 is considered to be of statistical significance.

**Results**

**Identification and Annotation of genes related to TNBC-specific glucose metabolism**

Gene expression profiles and SUV assessments of 84 TNBC patients were obtained from the GEO database (GSE135565). Patients were divided into either glucose uptake high (GUH) group (SUV≥4.6) or glucose uptake low (GUL) group (SUV＜4.6).^14^ WGCNA (Weighted gene co-expression network analysis) was conducted to identify differential gene modules between two groups. Genes with similar expression peculiarities established the same module (Figure 1A). The soft threshold β value equaled 18 when the co-expression network satisfied scale-free topology (Figure 1B). Six major gene modules were constructed using the average linkage hierarchical clustering (Figure 1C). Their correlations with SUVs were evaluated (Figure 1D). The upregulation of genes within the blue module has the strongest positive correlation with the GUH group. On the contrary, genes in module black have a significant relationship with the GUL group.

In these gene modules, the hub genes referred to those with MM >0.3 and GS >0.3. As a result, 246 in the blue modules (Figure 1E, Table S1) and 180 genes in the black modules (Figure 1F, Table S2) were revealed as the hub genes and included for further analysis.

To better depict these hub genes' biologic activity, the GO and KEGG enrichment analysis were conducted. The GO analyses revealed that the dominant biological functions include extracellular structure organization, cell-substrate adhesion, and positive regulation of wound healing. Meanwhile, these genes were involved in responses to transforming growth factor-beta and steroid hormone and the negative regulation of the immune effector process. Interestingly, these genes also play a role in the biological reaction towards different oxygen levels. Several genes were related to response to oxygen levels, cellular response to oxidative stress, and response hypoxia (Figure 2A). For KEGG analysis, pathways concerning extracellular matrix (ECM) regulation (ECM-receptor interaction, focal adhesion, and proteoglycans in cancer), nutrition regulation (protein digestion and absorption), and oncogenesis (PI3K-Akt signaling pathway) were closely related to the hub genes (Figure 2B). Surprisingly, the HPV (human papillomavirus) infection pathway is one of the most involved pathways. The HPV infection pathway is not only related to oncogenesis but also cancer-specific immune evasion. What’s more, the anti-tumor immune pathways can also be activated by antiviral signals^21^.

We depicted PPI networks of hub genes via the online tool STRING (URL: https://string-db.org/) to achieve a clear vision of the interactions among hub genes (Figure S1). The threshold of interaction score for high confidence was set at 0.7, and disconnected nodes in the network were hidden. A total of 165 hub genes were involved in the construction of this regulatory network. Correspondent proteins of hub genes have a relatively high internal connections level, indicating a firm inter-module relationship. Notably, FN1, a gene involved in cell adhesion and migration processes, including embryogenesis, wound healing, blood coagulation, host defense, and metastasis, appears to be the center of the PPI network. Previous studies suggested that FN1 is a major ECM component secreted by various epithelial or mesenchymal cell types in both physiologic and pathophysiologic situations in response to high glucose stimulations.^22-24^

**Reveal of the** **overlap between TNBC specific immunity and** **glucose metabolism**

For the next step, we focus on revealing the overlap between the above genes and cancer-specific immunity. Genes related to PD-(L)1 expression was identified using the RNA profiles of 146 TNBC and 111 normal tissues retrieved from the TCGA database. 3288 upregulated and 2914 down-regulated differentially expressed genes are identified (Figure 3A). Ten major gene modules were established by further WGCNA (Figure 3B). Genes within the brown module have the strongest positive correlation with the increment of PD-(L)1 in BC (Figure 3C). Thus, 329 genes in the brown module with MM >0.3 and GS >0.3 were regarded as hub genes (Figure 3D, 3E, and Table S3).

Most important, IL4I1, ITGB7, and FUT7 were revealed in both the cancer-immune gene set and glucose metabolic gene set (Figure 3F). They all had a moderate to strong positive relationship with the increased SUV and PD-(L)1 expression (Figure 4). These genes might be the potential linkage between cancer-specific immunity and glucose metabolism and were included for further analysis.

**The expression profile of** **IL4I1, ITGB7, and FUT7**

Recent advances in genome-wide gene expression profiling analyses improved our understanding of TNBC and revealed at least six different molecular subtypes of TNBC, namely Basal-like 1 (BL1), basal-like 2 (BL2), immunomodulatory (IM), mesenchymal (M), mesenchymal stem-like (MSL) and luminal androgen receptor (LAR).^25^ We explored the mRNA expressions of hub genes in these molecular subtypes of TNBC using UALCAN (http://ualcan.path.uab.edu), which was an interactive online database to perform in-depth analyses of gene expression data from TCGA.^26^ We found that all three genes were highly expressed in the IM subtype of TNBC (Figure 5). The IM subtype was characterized by significant gene enrichment in immune cell signaling, cytokine signaling, antigen processing and presentation, and signaling through core immune signal transduction pathways.

Similar results were revealed by the single-gene GSEA analysis (Figure 6). A high expression of IL4L1 was related to pathways of NK cell mediated cytotoxicity, T cell receptor signaling pathway, and antigen processing and presentation. The representative pathways associated with ITGB7, including cell adhesion molecules (CAMs), chemokine signaling pathway, and Fc gamma R mediated phagocytosis. FUT7 exhibited a potent relationship with the chemokine signaling pathway, cytokine cytokine-receptor interaction, and T cell receptor signaling pathway.

**Immune Cell Infiltration Analysis**

The relationship of IL4I1, ITGB7, and FUT7 and immune cell infiltration characterizations were further explored using TIMER databases (Figure 7). The expression of IL4I1 correlated with the infiltrating levels of B cells, CD4+ T cells, neutrophils, and DC cells positively. Similarly, ITGB7 and FUT7 were both positively correlated with the infiltrating levels of B cells, CD8+ T cells, CD4+ T cells, neutrophils, and DC cells. Most importantly, analysis of TIMER and GEPIA databases revealed that IL4I1, ITGB7, and FUT7 all have potent positive associations with the abundance of Treg cells, indicated their immunosuppressive effects (Figure S2, Table 1). Similar immune cell infiltration characterizations were found in the other two types (Luminal and HER2 positive) breast cancer (Figure S3). Furthermore, we observed a significant positive correlation between the expression of three hub genes and the expression of exhausted T cell markers, including LAG-3, TIM-3, CTLA-4, and CXCL13. Meanwhile, the levels of IL4I1, ITGB7, and FUT7 expressions also correlate with the expression of marker genes for TAM, M2, Tfh, and Th1 cell infiltration (Table 1).

**Prognosis analysis**

We evaluate the prognosis value of IL4I1, ITGB7 and FUT7 in TNBC by using PrognoScan (Table S4). Results showed that all of them were potent negative prognostic factors for OS (Overall Survival) (HR: 1.49 [1.02 - 2.18], *p*= 0.041; 1.85 [1.23 - 2.78] *p*= 0.003; 2.07 [1.08 - 3.97] *p*= 0.028, for IL4I1, ITGB7, and FUT7, respectively). IL4I1 and ITGB7 are also negative predictors for relapse (HR: 1.86 [1.25 - 2.76], *p*= 0.002; 4.41 [1.07 - 18.25], *p*= 0.041 for IL4I1 and ITGB7, respectively). GSE1456, GSE9195, GSE9893, GSE9195 and GSE3143 were involved in above survival analysis.

**Discussion**

This study provides novel insights into the linkage between two important hallmarks in cancer, glucose metabolism, and cancer-specific immunity. IL4I1, ITGB7, and FUT7, which were identified by comprehensive bioinformatic analysis, may play a vital role in the tumor immune microenvironment and hold the potential of enhancing ICB treatment by remodeling glucose metabolism. This hypothesis was also confirmed by gene enrichment analysis.

In this study, the glucose uptake intensities were presented through SUV assessed by FDG-PET. FDG-PET is a well-recognized imaging tool in reflecting glucose metabolism, and SUV is an objective indicator of glucose uptake.^27^ Compared with normal tissue, tumors often demonstrate an increased FDG-PET signal, which reflects a high rate of radiotracer uptake through membrane glucose transporters, phosphorylation by one of several hexokinase enzymes, and the resultant intracellular trapping of the radiotracer, which is not further metabolized in the cell.^28,29^ Indeed, there are concerns since the genomic characteristics of SUV have not been fully explored, and the validity of using SUV to reflect the complex effects of glucose metabolism on cancer progression is questionable and inaccurate. However, to date, FDG-PET is the only reflection of cancer metabolic behavior actually occurring in the human body.^30^ Meanwhile, plenty of previous studies have proved that SUV evaluated by FDG-PET is highly clinically and pathologically relevant.^31,32^

Moreover, the expression level of PD-(L)1 is used as a biomarker for cancer-specific immunity in our study, in accordance with previous studies, since they were the most important immune checkpoints and well-known therapeutic targets.^33-35^ We believe that genes related to PD-(L)1 hold the most substantial potential for future clinical application.

IL4I1, ITGB7, and FUT7 were identified in this study as key genes. In previous studies, they were considered to be important modifiers in tumor immune microenvironment and metabolism. IL4I1 is a bona fide immunosuppressive enzyme participating in the escape of malignancies in vivo.^36^ It accelerates tumor growth by limiting the CD8 T cell-mediated immune response in a mouse model of melanoma cell transplantation.^37,38^ The immune regulatory functions of IL4I1 have been attributed to either the depletion of amino acids, the promotion of Treg differentiation, or the upregulation of PD-1.^39,40^ What’s more, a recent study demonstrated that IL4I1 is a major aryl hydrocarbon receptor (AHR)-activating enzyme and endowed cancer cells with the capacity of migration and metastasis.^41^ ITGB7 is a key factor in the ITGB7/C/EBPβ signaling axis and integrin β4/FAK/SOX2/HIF-1α signaling pathway, which plays a vital part in glycolysis-induced cell growth and proliferation.^42,43^ The high involvement of C/EBPβ signaling and HIF-1α signaling pathway with immune inhabitation was also well-established.^44^ Meanwhile, Fucosyltransferase VII (FUT7) is one of the a1,3-fucosyltransferases family that catalyzes the final fucosylation step in generating a unique glycosylated product sialyl Lewis X (sLeX).^45,46^ sLeX can serve as ligands for E- or P-selectin expressed on the cell surface and results in cancer metastasis and angiogenesis.^11,46^ Additionally, increased fucosylated activity was also reported positively relative to increased glucose uptake by the pancreatic, lung, and ovarian carcinoma cell lines.^47^ According to previous studies, the upregulation of IL4I1 maybe a result of naturally occurring Single Nucleotide Polymorphisms (SNPs)^48^, the DNA demethylation of FUT7 could lead to its overexpression^49^, and highly expressed ITGB7 may be induced by TGF-beta1^50,51^. However, the potential basis for upregulation of above genes remains unknow in patients with breast cancers.

One of the most interesting findings observed in this study is the positive relationship between selected genes and the abundance of T cells, especially for CD4+ T cells and Treg cells. The mechanism of this connection is unclear. We assume that increased Treg cell infiltration may be the reason for inadequate responses to ICB treatment in TNBC, since Treg cells display functional instability and heterogeneity, and increased Treg frequency is often associated with reduced activity of antitumor cytotoxic T lymphocytes and worse prognosis^52^. The strong correlation between the expression of hub genes identified and the markers of T cell exhaustion may also exacerbate this situation. However, this hypothesis should be investigated in future studies.

There are still some limitations in this study. The major limitation of this study is that only *in silico* analysis were conducted. Therefore, all study results should be confirmed by *in vivo* or *in vitro* experiments in the future. Second, this study only applied analysis on the mRNA expression level. Thus, multi-omics analyses are warranted in the future.

In conclusion, IL4I1, ITGB7, and FUT7 were identified as the hub genes between two hallmarks in cancer, glucose metabolism, and cancer-specific immunity via comprehensive bioinformatic analysis. The GO and pathway enrichment analysis suggest that they may be potential targets for enhancing ICB treatment by reprogramming the tumor microenvironment and remodeling tumor metabolism.

**Declaration of Interest**

The authors declare that they have no conflict of interests.

**Acknowledgments**

The present work was supported by the National Natural Science Foundation of China (81902933).

**Author Contribution**

Q.G., X.L., and T.X. designed and concepted this study. T.X., J.L., and Y.X. collected, analyzed, and interpreted the data. J.L. wrote the manuscript. Q.G., Y.X., and Z.W. participated in revising the manuscript. Q.G. and X.L. supervised the whole project. All authors read and approved the final manuscript for publication.

**Data availability statement**

The data that support the findings of this study are openly available in Gene Expression Omnibus (GEO) database, GSE135565 dataset (http://www.ncbi.nlm.nih.gov/geo/).

**Reference**

1.	Bianchini G, Balko JM, Mayer IA, Sanders ME, Gianni L. Triple-negative breast cancer: challenges and opportunities of a heterogeneous disease. *Nature Reviews Clinical Oncology* 2016; **13**(11): 674-90.

2.	Foulkes WD, Smith IE, Reis-Filho JS. Triple-Negative Breast Cancer. *N Engl J Med* 2010; **363**(20): 1938-48.

3.	Pardoll DM. The blockade of immune checkpoints in cancer immunotherapy. *Nat Rev Cancer* 2012; **12**(4): 252-64.

4.	Adams S, Gatti-Mays ME, Kalinsky K, Korde LA, Sharon E, Amiri-Kordestani L, Bear H, McArthur HL, Frank E, Perlmutter J, et al. Current Landscape of Immunotherapy in Breast Cancer: A Review. *JAMA oncology* 2019; **5**(8): 1205-14.

5.	Vranic S, Cyprian FS, Gatalica Z, Palazzo J. PD-L1 status in breast cancer: Current view and perspectives. *Seminars in cancer biology* 2019.

6.	Chang C-H, Qiu J, O’Sullivan D, Buck Michael D, Noguchi T, Curtis Jonathan D, Chen Q, Gindin M, Gubin Matthew M, van der Windt Gerritje JW, et al. Metabolic Competition in the Tumor Microenvironment Is a Driver of Cancer Progression. *Cell* 2015; **162**(6): 1229-41.

7.	Brand A, Singer K, Koehl GE, Kolitzus M, Schoenhammer G, Thiel A, Matos C, Bruss C, Klobuch S, Peter K, et al. LDHA-Associated Lactic Acid Production Blunts Tumor Immunosurveillance by T and NK Cells. *Cell Metab* 2016; **24**(5): 657-71.

8.	Boussiotis VA. Molecular and Biochemical Aspects of the PD-1 Checkpoint Pathway. *N Engl J Med* 2016; **375**(18): 1767-78.

9.	Li CW, Lim SO, Chung EM, Kim YS, Park AH, Yao J, Cha JH, Xia W, Chan LC, Kim T, et al. Eradication of Triple-Negative Breast Cancer Cells by Targeting Glycosylated PD-L1. *Cancer Cell* 2018; **33**(2): 187-201 e10.

10.	Li CW, Lim SO, Xia W, Lee HH, Chan LC, Kuo CW, Khoo KH, Chang SS, Cha JH, Kim T, et al. Glycosylation and stabilization of programmed death ligand-1 suppresses T-cell activity. *Nat Commun* 2016; **7**: 12632.

11.	Sun L, Li CW, Chung EM, Yang R, Kim YS, Park AH, Lai YJ, Yang Y, Wang YH, Liu J, et al. Targeting Glycosylated PD-1 Induces Potent Antitumor Immunity. *Cancer Res* 2020; **80**(11): 2298-310.

12.	Martinez-Outschoorn UE, Peiris-Pagés M, Pestell RG, Sotgia F, Lisanti MP. Cancer metabolism: a therapeutic perspective. *Nature Reviews Clinical Oncology* 2017; **14**(1): 11-31.

13.	Gong Y, Ji P, Yang Y-S, Xie S, Yu T-J, Xiao Y, Jin M-L, Ma D, Guo L-W, Pei Y-C, et al. Metabolic-Pathway-Based Subtyping of Triple-Negative Breast Cancer Reveals Potential Therapeutic Targets. *Cell Metabolism* 2020.

14.	Kim SK, Ahn SG, Mun JY, Jeong MS, Bae SJ, Lee JS, Jeong J, Leem SH, Chu IS. Genomic Signature of the Standardized Uptake Value in (18)F-Fluorodeoxyglucose Positron Emission Tomography in Breast Cancer. *Cancers (Basel)* 2020; **12**(2).

15.	Kang S, Kim EH, Hwang JE, Shin JH, Jeong YS, Yim SY, Joo EW, Eun YG, Lee DJ, Sohn BH, et al. Prognostic significance of high metabolic activity in breast cancer: PET signature in breast cancer. *Biochem Biophys Res Commun* 2019; **511**(1): 185-91.

16.	Langfelder P, Horvath S. WGCNA: an R package for weighted correlation network analysis. *BMC Bioinformatics* 2008; **9**: 559.

17.	Li T, Wernersson R, Hansen RB, Horn H, Mercer J, Slodkowicz G, Workman CT, Rigina O, Rapacki K, Stærfeldt HH, et al. A scored human protein-protein interaction network to catalyze genomic interpretation. *Nat Methods* 2017; **14**(1): 61-4.

18.	Mizuno H, Kitada K, Nakai K, Sarai A. PrognoScan: a new database for meta-analysis of the prognostic value of genes. *BMC medical genomics* 2009; **2**: 18.

19.	Li T, Fan J, Wang B, Traugh N, Chen Q, Liu JS, Li B, Liu XS. TIMER: A Web Server for Comprehensive Analysis of Tumor-Infiltrating Immune Cells. *Cancer Res* 2017; **77**(21): e108-e10.

20.	Gu Y, Li X, Bi Y, Zheng Y, Wang J, Li X, Huang Z, Chen L, Huang Y, Huang Y. CCL14 is a prognostic biomarker and correlates with immune infiltrates in hepatocellular carcinoma. *Aging* 2020; **12**(1): 784-807.

21.	Bowling EA, Wang JH, Gong F, Wu W, Neill NJ, Kim IS, Tyagi S, Orellana M, Kurley SJ, Dominguez-Vidaña R, et al. Spliceosome-targeted therapies trigger an antiviral immune response in triple-negative breast cancer. *Cell* 2021.

22.	Saik OV, Klimontov VV. Bioinformatic Reconstruction and Analysis of Gene Networks Related to Glucose Variability in Diabetes and Its Complications. *Int J Mol Sci* 2020; **21**(22).

23.	Zhang H, Wang JW, Xu Y, Zhang K, Yi B, Sun J, Liu Y, Zhang XM, Liu JS. Effect of β-(3,4-dihydroxyphenyl)lactic acid on oxidative stress stimulated by high glucose levels in human peritoneal mesothelial cells. *The Journal of international medical research* 2012; **40**(3): 943-53.

24.	Alvarez ML, DiStefano JK. Functional characterization of the plasmacytoma variant translocation 1 gene (PVT1) in diabetic nephropathy. *PLoS One* 2011; **6**(4): e18671.

25.	Lehmann BD, Bauer JA, Chen X, Sanders ME, Chakravarthy AB, Shyr Y, Pietenpol JA. Identification of human triple-negative breast cancer subtypes and preclinical models for selection of targeted therapies. *The Journal of clinical investigation* 2011; **121**(7): 2750-67.

26.	Chandrashekar DS, Bashel B, Balasubramanya SAH, Creighton CJ, Ponce-Rodriguez I, Chakravarthi B, Varambally S. UALCAN: A Portal for Facilitating Tumor Subgroup Gene Expression and Survival Analyses. *Neoplasia (New York, NY)* 2017; **19**(8): 649-58.

27.	Ahn SG, Park JT, Lee HM, Lee HW, Jeon TJ, Han K, Lee SA, Dong SM, Ryu YH, Son EJ, et al. Standardized uptake value of 18F-fluorodeoxyglucose positron emission tomography for prediction of tumor recurrence in breast cancer beyond tumor burden. *Breast Cancer Research* 2014; **16**(6): 502.

28.	Jadvar H, Alavi A, Gambhir SS. 18F-FDG Uptake in Lung, Breast, and Colon Cancers: Molecular Biology Correlates and Disease Characterization. *J Nucl Med* 2009; **50**(11): 1820-7.

29.	Palaskas N, Larson SM, Schultz N, Komisopoulou E, Wong J, Rohle D, Campos C, Yannuzzi N, Osborne JR, Linkov I, et al. 18F-Fluorodeoxy-glucose Positron Emission Tomography Marks MYC-Overexpressing Human Basal-Like Breast Cancers. *Cancer Res* 2011; **71**(15): 5164-74.

30.	Fletcher JW, Djulbegovic B, Soares HP, Siegel BA, Lowe VJ, Lyman GH, Coleman RE, Wahl R, Paschold JC, Avril N, et al. Recommendations on the use of 18F-FDG PET in oncology. *J Nucl Med* 2008; **49**(3): 480-508.

31.	Shimoda W, Hayashi M, Murakami K, Oyama T, Sunagawa M. The relationship between FDG uptake in PET scans and biological behavior in breast cancer. *Breast cancer (Tokyo, Japan)* 2007; **14**(3): 260-8.

32.	Ahn SG, Lee J-H, Lee HW, Jeon TJ, Ryu YH, Kim KM, Sohn J, Yun M, Lee SA, Jeong J, et al. Comparison of standardized uptake value of 18F-FDG-PET-CT with 21-gene recurrence score in estrogen receptor-positive, HER2-negative breast cancer. *PLOS ONE* 2017; **12**(4): e0175048.

33.	Xu T, Wang Z, Dong M, Wu D, Liao S, Li X. Chloride intracellular channel protein 2: prognostic marker and correlation with PD-1/PD-L1 in breast cancer. *Aging* 2020; **12**(17): 17305-27.

34.	Tomioka N, Azuma M, Ikarashi M, Yamamoto M, Sato M, Watanabe KI, Yamashiro K, Takahashi M. The therapeutic candidate for immune checkpoint inhibitors elucidated by the status of tumor-infiltrating lymphocytes (TILs) and programmed death ligand 1 (PD-L1) expression in triple negative breast cancer (TNBC). *Breast cancer (Tokyo, Japan)* 2018; **25**(1): 34-42.

35.	Park IH, Kong SY, Ro JY, Kwon Y, Kang JH, Mo HJ, Jung SY, Lee S, Lee KS, Kang HS, et al. Prognostic Implications of Tumor-Infiltrating Lymphocytes in Association With Programmed Death Ligand 1 Expression in Early-Stage Breast Cancer. *Clinical breast cancer* 2016; **16**(1): 51-8.

36.	Lasoudris F, Cousin C, Prevost-Blondel A, Martin-Garcia N, Abd-Alsamad I, Ortonne N, Farcet J-P, Castellano F, Molinier-Frenkel V. IL4I1: an inhibitor of the CD8+ antitumor T-cell response in vivo. *European Journal of Immunology* 2011; **41**(6): 1629-38.

37.	Bod L, Lengagne R, Wrobel L, Ramspott JP, Kato M, Avril MF, Castellano F, Molinier-Frenkel V, Prevost-Blondel A. IL4-induced gene 1 promotes tumor growth by shaping the immune microenvironment in melanoma. *Oncoimmunology* 2017; **6**(3): e1278331.

38.	Yue Y, Huang W, Liang J, Guo J, Ji J, Yao Y, Zheng M, Cai Z, Lu L, Wang J. IL4I1 Is a Novel Regulator of M2 Macrophage Polarization That Can Inhibit T Cell Activation via L-Tryptophan and Arginine Depletion and IL-10 Production. *PLoS One* 2015; **10**(11): e0142979.

39.	Molinier-Frenkel V, Prévost-Blondel A, Castellano F. The IL4I1 Enzyme: A New Player in the Immunosuppressive Tumor Microenvironment. *Cells* 2019; **8**(7).

40.	Mezrich JD, Fechner JH, Zhang X, Johnson BP, Burlingham WJ, Bradfield CA. An interaction between kynurenine and the aryl hydrocarbon receptor can generate regulatory T cells. *J Immunol* 2010; **185**(6): 3190-8.

41.	Sadik A, Somarribas Patterson LF, Ozturk S, Mohapatra SR, Panitz V, Secker PF, Pfander P, Loth S, Salem H, Prentzell MT, et al. IL4I1 Is a Metabolic Immune Checkpoint that Activates the AHR and Promotes Tumor Progression. *Cell* 2020; **182**(5): 1252-70 e34.

42.	Chai Z, Yang Y, Gu Z, Cai X, Ye W, Kong L, Qiu X, Ying L, Wang Z, Wang L. Recombinant Viral Capsid Protein L2 (rVL2) of HPV 16 Suppresses Cell Proliferation and Glucose Metabolism via ITGB7/C/EBPbeta Signaling Pathway in Cervical Cancer Cell Lines. *Onco Targets Ther* 2019; **12**: 10415-25.

43.	Gan L, Meng J, Xu M, Liu M, Qi Y, Tan C, Wang Y, Zhang P, Weng W, Sheng W, et al. Extracellular matrix protein 1 promotes cell metastasis and glucose metabolism by inducing integrin beta4/FAK/SOX2/HIF-1alpha signaling pathway in gastric cancer. *Oncogene* 2018; **37**(6): 744-55.

44.	Cha JH, Chan LC, Li CW, Hsu JL, Hung MC. Mechanisms Controlling PD-L1 Expression in Cancer. *Mol Cell* 2019; **76**(3): 359-70.

45.	Liu S, Zhang Y, Liu Y, Qin H, Wang X, Yan Q. FUT7 antisense sequence inhibits the expression of FUT7/sLeX and adhesion between embryonic and uterine cells. *IUBMB life* 2008; **60**(7): 461-6.

46.	Liang JX, Gao W, Cai L. Fucosyltransferase VII promotes proliferation via the EGFR/AKT/mTOR pathway in A549 cells. *Onco Targets Ther* 2017; **10**: 3971-8.

47.	Babiuch K, Dag A, Zhao J, Lu H, Stenzel MH. Carbohydrate-Specific Uptake of Fucosylated Polymeric Micelles by Different Cancer Cell Lines. *Biomacromolecules* 2015; **16**(7): 1948-57.

48.	Molinier-Frenkel V, Mestivier D, Castellano F. Alterations of the immunosuppressive IL4I1 enzyme activity induced by naturally occurring SNP/mutations. *Genes and immunity* 2016; **17**(2): 148-52.

49.	Pink M, Ratsch BA, Mardahl M, Durek P, Polansky JK, Karl M, Baumgrass R, Wallner S, Cadenas C, Gianmoena K, et al. Imprinting of Skin/Inflammation Homing in CD4+ T Cells Is Controlled by DNA Methylation within the Fucosyltransferase 7 Gene. *J Immunol* 2016; **197**(8): 3406-14.

50.	Wang J, Xiang H, Lu Y, Wu T. Role and clinical significance of TGF‑β1 and TGF‑βR1 in malignant tumors (Review). *International journal of molecular medicine* 2021; **47**(4).

51.	Lim SP, Leung E, Krissansen GW. The beta7 integrin gene (Itgb-7) promoter is responsive to TGF-beta1: defining control regions. *Immunogenetics* 1998; **48**(3): 184-95.

52.	Togashi Y, Shitara K, Nishikawa H. Regulatory T cells in cancer immunosuppression - implications for anticancer therapy. *Nature reviews Clinical oncology* 2019; **16**(6): 356-71.

Table 1. Correlation analysis between IL4I1, ITGB7	, and FUT7 and relate genes and markers of immune cells in GEPIA2.

| Description | Gene markers | IL4I1 | | | ITGB7 | | | FUT7 | |
| --- | --- | --- | --- | --- | --- | --- | --- | --- | --- |
|  |  | Core | P | Core | | P | Core | | P |
| T cell (general) | CD3D | 0.63 | 0.000 | 0.73 | | 0.000 | 0.79 | | 0.000 |
|  | CD3E | 0.60 | 0.000 | 0.73 | | 0.000 | 0.76 | | 0.000 |
|  | CD2 | 0.64 | 0.000 | 0.74 | | 0.000 | 0.75 | | 0.000 |
| TAM | CCL2 | 0.55 | 0.000 | 0.51 | | 0.000 | 0.49 | | 0.000 |
|  | CD68 | 0.67 | 0.000 | 0.50 | | 0.000 | 0.46 | | 0.000 |
|  | IL10 | 0.59 | 0.000 | 0.52 | | 0.000 | 0.44 | | 0.000 |
| M1 Macrophage | iNOS | - | - | - | | - | - | | - |
|  | IRF5 | 0.41 | 0.000 | 0.43 | | 0.000 | 0.43 | | 0.000 |
|  | COX2 | 0.13 | 0.000 | 0.22 | | 0.000 | 0.20 | | 0.000 |
| M2 Macrophage | CD163 | 0.70 | 0.000 | 0.56 | | 0.000 | 0.55 | | 0.000 |
|  | VSIG4 | 0.47 | 0.000 | 0.39 | | 0.000 | 0.40 | | 0.000 |
|  | MS4A4A | 0.58 | 0.000 | 0.52 | | 0.000 | 0.50 | | 0.000 |
| Tfh | BCL6 | - | - | - | | - | - | | - |
|  | IL21 | 0.49 | 0.000 | 0.55 | | 0.000 | 0.49 | | 0.000 |
| Th1 | T-bet | 0.63 | 0.000 | 0.74 | | 0.000 | 0.76 | | 0.000 |
|  | STAT4 | 0.51 | 0.000 | 0.64 | | 0.000 | 0.64 | | 0.000 |
|  | STAT1 | 0.55 | 0.000 | 0.51 | | 0.000 | 0.38 | | 0.000 |
|  | IFN-γ | 0.61 | 0.000 | 0.65 | | 0.000 | 0.62 | | 0.000 |
|  | TNF-α | 0.49 | 0.000 | 0.41 | | 0.000 | 0.43 | | 0.000 |
| Th2 | GATA3 | -0.38 | 0.000 | -0.29 | | 0.000 | -0.29 | | 0.000 |
|  | STAT6 | - | - | - | | - | - | | - |
|  | STAT5A | 0.21 | 0.000 | 0.29 | | 0.000 | 0.32 | | 0.000 |
|  | IL13 | 0.29 | 0.000 | 0.29 | | 0.000 | 0.28 | | 0.000 |
| Th17 | STAT3 | - | - | - | | - | - | | - |
|  | IL17A | 0.27 | 0.000 | 0.27 | | 0.000 | 0.25 | | 0.000 |
| Treg | FOXP3 | 0.68 | 0.000 | 0.65 | | 0.000 | 0.66 | | 0.000 |
|  | CCR8 | 0.59 | 0.000 | 0.54 | | 0.000 | 0.50 | | 0.000 |
|  | TGFβ | 0.30 | 0.000 | 0.26 | | 0.000 | 0.41 | | 0.000 |
| T cell exhaustion | CTLA4 | 0.72 | 0.000 | 0.72 | | 0.000 | 0.70 | | 0.000 |
|  | LAG3 | 0.69 | 0.000 | 0.66 | | 0.000 | 0.64 | | 0.000 |
|  | TIM-3 | 0.66 | 0.000 | 0.50 | | 0.000 | 0.50 | | 0.000 |
|  | CXCL13 | 0.48 | 0.000 | 0.50 | | 0.000 | 0.55 | | 0.000 |

TAM, tumor-associated macrophage; Th, T helper cell; Tfh, Follicular helper T cell; Treg, regulatory T cell; Cor, R value of Spearman’s correlation. “-” represents |R|＜0.1.

**Figures**

Figure 1. Identification of genes related to TNBC-specific glucose metabolism.

Figure 2. GO and KEGG enrichment analysis for metabolism-related hub genes.

Figure 3. Identification of immune-related genes in TNBC.

Figure 4. Correlation between hub gene expression and SUV and PD-(L)1 expression.

Figure 5. Expression levels of IL4I1 (A, B), ITGB7 (C, D), and FUT7 (E, F) in different breast cancer subtypes or TNBC subtypes.

Figure 6. GSEA analysis of TNBC grouped by IL4I1, ITGB7, and FUT7 expression.

Figure 7. Immune cell infiltration analysis.

**Figure captions**

Figure 1. Identification of genes related to TNBC-specific glucose metabolism.

(A) Sample clustering to detect outliers. (B) Determination of soft threshold by evaluating the scale-free topology fit index (left) and mean connectivity (right). (C) Dendrogram plot with color annotation. (D) Heatmap for the correlations of gene modules to SUV. Correlations between module membership and gene significance values were presented in scatterplots for the blue (E) and black (F) gene modules. TNBC, triple-negative breast cancer; SUV, standardized uptake value.

Figure 2. GO and KEGG enrichment analysis for metabolism-related hub genes.

(A) Major GO enrichment terms in hub gene clusters correlated to metabolism; (B) KEGG pathway enrichment in hub gene clusters correlated to metabolism. GO, Gene Ontology; KEGG, Kyoto Encyclopedia of Genes and Genomes.

Figure 3. Identification of immune-related genes in TNBC.

(A) Volcano plot showing differentially expressed genes. (B) Dendrogram plot with color annotation. (C) Heatmap for the correlations of gene modules to PD-(L)1. Scatterplots for correlations between module membership and gene significance values concerning PD-1 (D) and PD-L1 (E) expression for the brown gene module. (F) Venn diagram of overlapping genes identified by the immune-related gene set and metabolic gene set. TNBC, triple-negative breast cancer.

Figure 4. Correlation between hub gene expression and SUV and PD-(L)1 expression.

The Spearman correlation coefficients between IL4I1 (A to C), ITGB7 (D to F), and FUT7 (G to I) expression and SUV, PD-1, and PD-L1 levels were shown. SUV, standardized uptake value.

Figure 5. Expression levels of IL4I1 (A, B), ITGB7 (C, D), and FUT7 (E, F) in different breast cancer subtypes or TNBC subtypes. TNBC, triple-negative breast cancer.

Figure 6. GSEA analysis of TNBC grouped by IL4I1, ITGB7, and FUT7 expression. GSEA was performed on RNA-Seq data of 146 TNBC samples grouped by IL4I1, ITGB7, and FUT7 expression levels, respectively. Representative pathways of IL4I1 (A to C), ITGB7 (D to F), and FUT7 (G to I) were shown. GSEA, gene set enrichment analysis.

Figure 7. Immune cell infiltration analysis. The TIMER was used to evaluate the correlations between immune cell abundance and IL4I1 (A), ITGB7 (B), and FUT7 (C) expressions in basal breast cancer. TIMER, Tumor Immune Estimation Resource.

**Supplementary_Materials**

**Supplementary Tables**

Table S1. A total of 246 glucose uptake-related hub genes in blue module were identified by WGCNA (top 20 hub genes with the highest GS.High scores were shown).

| probes | Module  Color | GS.Low | p.GS.Low | GS.High | p.GS.High | MM | p.MM |
| --- | --- | --- | --- | --- | --- | --- | --- |
| IL4I1 | blue | -0.47 | 5.29E-06 | 0.47 | 5.29E-06 | 0.75 | 2.68E-16 |
| B4GALT2 | blue | -0.44 | 2.23E-05 | 0.44 | 2.23E-05 | 0.79 | 3.16E-19 |
| CA9 | blue | -0.43 | 4.05E-05 | 0.43 | 4.05E-05 | 0.65 | 1.79E-11 |
| LINC00482 | blue | -0.42 | 7.40E-05 | 0.42 | 7.40E-05 | 0.77 | 1.08E-17 |
| AOAH | blue | -0.42 | 7.77E-05 | 0.42 | 7.77E-05 | 0.74 | 7.22E-16 |
| CHCHD1 | blue | -0.42 | 8.37E-05 | 0.42 | 8.37E-05 | 0.61 | 8.06E-10 |
| PRAM1 | blue | -0.41 | 0.000109 | 0.41 | 0.000109 | 0.82 | 1.30E-21 |
| SLC25A47 | blue | -0.41 | 0.000113 | 0.41 | 0.000113 | 0.70 | 2.01E-13 |
| ZP1 | blue | -0.41 | 0.000119 | 0.41 | 0.000119 | 0.72 | 1.87E-14 |
| APC2 | blue | -0.40 | 0.000139 | 0.40 | 0.000139 | 0.88 | 1.35E-27 |
| UPP1 | blue | -0.40 | 0.000149 | 0.40 | 0.000149 | 0.65 | 1.91E-11 |
| PNCK | blue | -0.40 | 0.000169 | 0.40 | 0.000169 | 0.84 | 5.43E-24 |
| ACTL10 | blue | -0.39 | 0.000223 | 0.39 | 0.000223 | 0.66 | 9.00E-12 |
| MMP17 | blue | -0.39 | 0.000240 | 0.39 | 0.000240 | 0.82 | 1.42E-21 |
| FBXL6 | blue | -0.39 | 0.000245 | 0.39 | 0.000245 | 0.75 | 1.61E-16 |
| TNNI1 | blue | -0.39 | 0.000246 | 0.39 | 0.000246 | 0.81 | 1.69E-20 |
| S1PR4 | blue | -0.39 | 0.000254 | 0.39 | 0.000254 | 0.85 | 2.17E-24 |
| POU6F2 | blue | -0.39 | 0.000255 | 0.39 | 0.000255 | 0.76 | 4.25E-17 |
| SURF2 | blue | -0.38 | 0.000304 | 0.38 | 0.000304 | 0.90 | 1.47E-30 |
| PLA2G2E | blue | -0.38 | 0.000326 | 0.38 | 0.000326 | 0.70 | 1.72E-13 |

GS score represents the correlation between the gene and the standardized uptake value, and the MM score denotes the correlation between the gene and its module.

Table S2. A total of 180 glucose uptake-related hub genes in black module were identified by WGCNA (top 20 hub genes with the lowest GS.High scores were shown).

| probes | Module  Color | GS.Low | | p.GS.Low | GS.High | p.GS.High | MM | p.MM |
| --- | --- | --- | --- | --- | --- | --- | --- | --- |
| OMD | black | 0.60 | 2.36E-09 | | -0.60 | 2.36E-09 | 0.73 | 3.01E-15 |
| FHL1 | black | 0.59 | 2.76E-09 | | -0.59 | 2.76E-09 | 0.77 | 1.03E-17 |
| EDNRA | black | 0.58 | 9.62E-09 | | -0.58 | 9.62E-09 | 0.87 | 2.57E-26 |
| TIMP3 | black | 0.57 | 1.61E-08 | | -0.57 | 1.61E-08 | 0.78 | 4.27E-18 |
| RGS5 | black | 0.56 | 2.45E-08 | | -0.56 | 2.45E-08 | 0.71 | 5.27E-14 |
| HTRA1 | black | 0.55 | 5.79E-08 | | -0.55 | 5.79E-08 | 0.82 | 6.29E-22 |
| CILP | black | 0.55 | 6.44E-08 | | -0.55 | 6.44E-08 | 0.79 | 3.21E-19 |
| CYBRD1 | black | 0.55 | 6.78E-08 | | -0.55 | 6.78E-08 | 0.84 | 5.48E-23 |
| ITGBL1 | black | 0.54 | 1.04E-07 | | -0.54 | 1.04E-07 | 0.77 | 8.57E-18 |
| GPRASP1 | black | 0.54 | 1.41E-07 | | -0.54 | 1.41E-07 | 0.65 | 3.06E-11 |
| EBF1 | black | 0.53 | 1.74E-07 | | -0.53 | 1.74E-07 | 0.84 | 8.78E-24 |
| RP11-524D16 | black | 0.53 | 1.84E-07 | | -0.53 | 1.84E-07 | 0.79 | 7.23E-19 |
| COLEC12 | black | 0.51 | 5.80E-07 | | -0.51 | 5.8E-07 | 0.79 | 2.59E-19 |
| CD36 | black | 0.51 | 6.25E-07 | | -0.51 | 6.25E-07 | 0.65 | 1.76E-11 |
| SEMA3C | black | 0.51 | 6.26E-07 | | -0.51 | 6.26E-07 | 0.70 | 1.45E-13 |
| PDGFD | black | 0.51 | 6.89E-07 | | -0.51 | 6.89E-07 | 0.77 | 2.22E-17 |
| ZBTB20 | black | 0.51 | 8.15E-07 | | -0.51 | 8.15E-07 | 0.69 | 2.45E-13 |
| OGN | black | 0.51 | 8.15E-07 | | -0.51 | 8.15E-07 | 0.80 | 1.03E-19 |
| SH3D19 | black | 0.51 | 9.47E-07 | | -0.51 | 9.47E-07 | 0.82 | 6.73E-22 |
| ZFP36 | black | 0.50 | 1.10E-06 | | -0.51 | 1.10E-06 | 0.67 | 2.02E-12 |

GS score represents the correlation between the gene and the standardized uptake value, and the MM score denotes the correlation between the gene and its module.

Table S3. A total of 329 PD-(L)1-related hub genes in brown module were identified by WGCNA (top 20 hub genes with the highest GS.PD-L1 scores were shown).

| probes | Module  Color | GS.PD-1 | | p.GS.PD-1 | GS.PD-L1 | p.GS.PD-  L1 | MM | p.MM |
| --- | --- | --- | --- | --- | --- | --- | --- | --- |
| IDO1 | brown | 0.60 | 4.94E-13 | | 0.88 | 4.20E-41 | 0.38 | 1.22E-05 |
| CD38 | brown | 0.79 | 5.00E-27 | | 0.86 | 1.88E-37 | 0.75 | 3.28E-23 |
| AL591468.1 | brown | 0.69 | 2.95E-18 | | 0.85 | 7.44E-36 | 0.56 | 1.35E-11 |
| GBP5 | brown | 0.63 | 9.36E-15 | | 0.85 | 1.21E-34 | 0.44 | 4.96E-07 |
| LILRB4 | brown | 0.66 | 1.18E-16 | | 0.84 | 4.79E-33 | 0.57 | 6.83E-12 |
| IFNG | brown | 0.73 | 3.62E-21 | | 0.83 | 4.88E-32 | 0.55 | 4.23E-11 |
| FASLG | brown | 0.76 | 2.89E-24 | | 0.82 | 1.46E-30 | 0.67 | 5.08E-17 |
| FCGR1B | brown | 0.69 | 1.80E-18 | | 0.80 | 8.81E-29 | 0.54 | 9.97E-11 |
| CXCR2P1 | brown | 0.71 | 6.11E-20 | | 0.80 | 7.91E-28 | 0.60 | 1.92E-13 |
| GBP1P1 | brown | 0.57 | 1.08E-11 | | 0.79 | 8.56E-28 | 0.41 | 2.70E-06 |
| ADAMDEC | brown | 0.62 | 1.87E-14 | | 0.79 | 1.42E-27 | 0.56 | 2.01E-11 |
| FCGR3A | brown | 0.59 | 1.48E-12 | | 0.78 | 1.57E-26 | 0.46 | 7.56E-08 |
| FCGR1A | brown | 0.65 | 2.79E-16 | | 0.78 | 1.67E-26 | 0.53 | 4.98E-10 |
| IRF1 | brown | 0.71 | 3.05E-20 | | 0.78 | 2.01E-26 | 0.64 | 2.82E-15 |
| LAG3 | brown | 0.76 | 4.18E-24 | | 0.78 | 3.79E-26 | 0.61 | 1.48E-13 |
| KCNJ10 | brown | 0.63 | 7.57E-15 | | 0.78 | 4.21E-26 | 0.52 | 5.44E-10 |
| LILRB1 | brown | 0.74 | 4.52E-22 | | 0.77 | 1.24E-25 | 0.76 | 6.54E-24 |
| GBP1 | brown | 0.59 | 5.23E-13 | | 0.76 | 3.36E-24 | 0.50 | 4.05E-09 |
| USP30-AS1 | brown | 0.76 | 1.59E-24 | | 0.76 | 4.14E-24 | 0.66 | 1.91E-16 |
| CXCR6 | brown | 0.85 | 1.26E-34 | | 0.75 | 3.08E-23 | 0.78 | 9.87E-26 |

GS score represents the correlation between the gene and the PD-(L)1 expression, and the MM score denotes the correlation between the gene and its module.

Table S4. Prognosis analysis for IL4I1, ITGB7, and FUT7 (the PrognoScan database).

| Gene | Dataset | Endpoint | Number | COX P-value | HR [95% CI-low CI-up] |
| --- | --- | --- | --- | --- | --- |
| IL4I1 | GSE1456 | RFS | 159 | 0.002 | 1.86 [1.25 - 2.76] |
| IL4I1 | GSE1456 | DSS | 159 | 0.005 | 1.95 [1.22 - 3.12] |
| IL4I1 | GSE9195 | RFS | 77 | 0.037 | 1.93 [1.04 - 3.59] |
| IL4I1 | GSE1456 | OS | 159 | 0.041 | 1.49 [1.02 - 2.18] |
| ITGB7 | GSE9893 | OS | 155 | 0.003 | 1.85 [1.23 - 2.78] |
| ITGB7 | GSE9195 | RFS | 77 | 0.041 | 4.41 [1.07 - 18.25] |
| FUT7 | GSE3143 | OS | 158 | 0.028 | 2.07 [1.08 - 3.97] |

RFS, relapse free survival; DSS, disease specific survival; OS, overall survival; HR, Hazard Ratio.

**Supplementary Figures**

Figure S1. PPI network for metabolism-related hub genes.

Figure S2. Tregs infiltration analysis.

Figure S3. Immune cell infiltration analysis.

**Supplementary figure captions**

Figure S1. PPI network for metabolism-related hub genes. (A) PPI networks were drawn by STRING (URL: https://string-db.org/). The minimum interaction score was set at 0.7. The disconnected nodes were not shown in the network. (B) The number of connections for hub genes. PPI, protein-protein interaction.

Figure S2. Tregs infiltration analysis. The TIMER was used to evaluate the correlations between Tregs abundance and IL4I1 (A), ITGB7 (B), and FUT7 (C) expressions in basal breast cancer. Tregs, regulatory T cells; TIMER, Tumor Immune Estimation Resource.

Figure S3. Immune cell infiltration analysis. The TIMER was used to evaluate the correlations between immune cell abundance and IL4I1 (A), ITGB7 (B), and FUT7 (C) expressions in Luminal and HER2 positive breast cancer. TIMER, Tumor Immune Estimation Resource.
